# ALI multilayered co-cultures mimic biochemical mechanisms of the cancer cell-fibroblast cross-talk involved in NSCLC MultiDrug Resistance

**DOI:** 10.1186/s12885-019-6038-x

**Published:** 2019-08-29

**Authors:** Dania Movia, Despina Bazou, Adriele Prina-Mello

**Affiliations:** 1 Department of Clinical Medicine/Trinity Translational Medicine Institute (TTMI), Trinity Centre for Health Sciences, University of Dublin Trinity College, James’s Street, D8, Dublin, Ireland; 2 Mater Misericordiae University Hospital, Dublin, Ireland; 3 AMBER Centre, CRANN Institute, University of Dublin Trinity College, Dublin, Ireland

**Keywords:** 3D in vitro model, Lung cancer, Inhaled drugs, ALI cultures

## Abstract

**Background:**

Lung cancer is the leading cause of cancer-related deaths worldwide. This study focuses on its most common form, Non-Small-Cell Lung Cancer (NSCLC). No cure exists for advanced NSCLC, and patient prognosis is extremely poor. Efforts are currently being made to develop effective inhaled NSCLC therapies. However, at present, reliable preclinical models to support the development of inhaled anti-cancer drugs do not exist. This is due to the oversimplified nature of currently available in vitro models, and the significant interspecies differences between animals and humans.

**Methods:**

We have recently established 3D Multilayered Cell Cultures (MCCs) of human NSCLC (A549) cells grown at the Air-Liquid Interface (ALI) as the first in vitro tool for screening the efficacy of inhaled anti-cancer drugs. Here, we present an improved in vitro model formed by growing A549 cells and human fibroblasts (MRC-5 cell line) as an ALI multilayered co-culture. The model was characterized over 14-day growth and tested for its response to four benchmarking chemotherapeutics.

**Results:**

ALI multilayered co-cultures showed an increased resistance to the four drugs tested as compared to ALI multilayered mono-cultures. The signalling pathways involved in the culture MultiDrug Resistance (MDR) were influenced by the cancer cell-fibroblast cross-talk, which was mediated through TGF-β1 release and subsequent activation of the PI3K/AKT/mTOR pathway. As per in vivo conditions, when inhibiting mTOR phosphorylation, MDR was triggered by activation of the MEK/ERK pathway activation and up-regulation in cIAP-1/2 expression.

**Conclusions:**

Our study opens new research avenues for the development of alternatives to animal-based inhalation studies, impacting the development of anti-NSCLC drugs.

**Electronic supplementary material:**

The online version of this article (10.1186/s12885-019-6038-x) contains supplementary material, which is available to authorized users.

## Background

With 353,000 deaths every year in Europe alone (~ 20% of total European cancer deaths (https://ec.europa.eu/eurostat/statistics-explained/index.php/Causes_of_death_statistics)), and with the number of affected patients growing larger every day in developing countries, lung cancer has become the leading cause of cancer-related deaths worldwide [1]. Early-stage lung cancer is often curable with surgery, but patients are rarely diagnosed at this stage due to the lack of clear symptoms. Patients with advanced (Stage IIIB) or metastatic (Stage IV) disease are offered therapy with only the aim of prolonging their survival, as no cure exists for Stage III/IV lung cancer. The prognosis is usually very poor for patients when diagnosed at these disease stages: seven out of eight die within the next 5 years [2]. Of these, 80% die within 1 year [2].

The current methods used to administer chemotherapeutics for lung cancer treatment (namely, intravenous injection or oral ingestion) play a significant role in why current treatments are relatively ineffective. Evidence supports the potential advantages of using inhalation rather than intravenous/oral drug administration routes in the treatment of respiratory diseases [3] such as lung cancer [4]. Despite suffering from poor lung deposition [5], which may cause inadequate patient compliance, inhalation allows for the administration of lower doses when compared with systemic delivery [6–8]. This is considered the main advantage of the inhalational route and it derives from the direct delivery of the active principle to the site-of-action and the avoidance of first-pass metabolism. This offers a faster onset of therapeutic action and minimizes the number and severity of the drug systemic adverse effects towards non-targeted organs and healthy cells [9, 10]. In addition, treatment regimens delivered via needle-free, non-invasive methods like inhalation, exhibit increased patient acceptance.

The clinical translation of inhaled cancer therapies is currently impaired by the complete lack of preclinical (in vitro or in vivo) models capable of predicting the behaviour and action of such drugs in human patients. The aim of this study is to facilitate such translation by establishing a novel in vitro co-culture model of Non-Small-Cell Lung Cancer (NSCLC) adenocarcinoma compatible with the efficacy screening of aerosol anti-cancer drugs. It should be noted that, NSCLC is the most frequent form of lung cancer, occurring in up to 85% of cases, and adenocarcinoma is its main subtype (incidence, ∼50%) [11].

In general, in vitro drug testing relies on submerged, two-dimensional (2D) cultures [12]. Submerged culture of NSCLC cells is possible; however, cells in this system do not reproduce the three-dimensional structure of the human tissue. To overcome this issue, complex 3D in vitro models have long been used in cancer research, to better mimic the architecture and function of human, heterogeneous tumour tissue [13]. Spherical 3D cell cultures are the most exploited in vitro model in cancer research [14], and spherical 3D models of NSCLC, such as tumour spheroids and multicellular tumoroids, have been previously described in the literature [15–17]. Such models allow co-culturing stromal and malignant cells with direct cell-to-cell contact. However, they lack direct contact of NSCLC cells with the gas phase (air). This point is particularly important for NSCLC adenocarcinoma, as the latter arises in the distal airways and is therefore exposed to the air. Also, recent findings have shown that Air-Liquid Interface (ALI) culturing conditions are essential for successfully mimicking the NSCLC pathogenesis in vitro [18]. ALI cultures are the only preclinical model allowing researchers to culture human NSCLC cells in an in vitro environment that incorporates the direct contact of cancer cells with air. In the ALI culture system, cells are seeded onto the semi-permeable membrane of hanging Transwell™ inserts and submerged in culture medium. Once the cells reach confluence, they are “air-lifted” by removing the medium from the upper chamber of the inserts and exposing the apical surface of the cells to the air. This system allows researchers to test the effects of aerosolized particles (including drug formulations) on the cells.

In order to establish a novel in vitro co-culture model of lung adenocarcinoma that enables the efficacy screening of aerosol chemotherapeutics, in the present study we developed a 3D ALI Multilayered Cell Culture (MCC) of human NSCLC cells (A549 cell line) co-cultured with human fibroblasts (MRC-5 cell line). In our most recent work [19], we have shown that it is possible to form an ALI MCC of A549 cells on Transwell™ inserts, making it compatible with the testing of aerosol chemotherapeutics administered by a clinical nebulizer. Nevertheless, the ALI MCC of A549 cells constitutes an oversimplified model of the human NSCLC tissue, as it lacks several components of the Tumour MicroEnvironment (TME) and, subsequently, various patient-relevant chemoresistance mechanisms. In the present study, we increased the complexity of the in vitro model previously reported by co-culturing human fibroblasts with tumour cells in order to represent one of the key TME cellular components promoting NSCLC progression and chemoresistance [20].

In the present work we first describe the formation and phenotypic properties of the ALI multilayered co-cultures developed, followed by the evaluation of their chemoresistance to four chemotherapeutic agents (namely, docetaxel, cytarabine, vinblastine and methotrexate). Our results show that MultiDrug Resistance (MDR) could be detected in ALI multilayered co-cultures. MDR was significantly higher in the co-cultures than in the ALI multilayered mono-culture model previously reported by the authors [19]. The molecular pathways activated by exposure to the four drugs demonstrated that the MDR mechanisms mimicked in vitro were strongly influenced by the cellular composition (mono- or multi-cellular) of the 3D culture itself. To support this conclusion, an in depth comparison with data previously published on ALI multilayered mono-cultures [19] is presented in the sections below.

## Methods

### Cell culture

Human adenocarcinoma cells (A549 cell line) and human lung fibroblasts (MRC-5 cell line; ATCC® CCL-171™; Lot. # 60000139) were obtained from the American Tissue Culture Collection (ATCC®) (LG Standards, England). The A549 cell line was successfully authenticated, as previously reported [19]. A549 cell line-specific phenotypic responses (e.g. p21 expression in response to DNA damage) were confirmed as part of the laboratory GLP. Such results are available in [19]. A549 cells were cultured in Dulbecco’s Modified Eagle Medium (DMEM) (Gibco, Invitrogen, Bio-Sciences Ltd., Ireland) supplemented with glucose (1,000 mg/l), gentamicin (5 μg/ml) and 10% Fetal Bovine Serum (FBS) (Sigma-Aldrich, Ireland). MRC-5 cells were cultured in Modified Eagle Medium (MEM) (Gibco, Invitrogen, Bio-Sciences Ltd., Ireland) supplemented with 1% penicillin/streptomycin (Gibco, Invitrogen, Bio-Sciences Ltd., Ireland) and 10% FBS. Cells were cultured at 37 °C and 5% CO_2_. For cell seeding, cells were detached from cell culture flasks’ substrate with TryplE™ (Gibco, Invitrogen, Bio-Sciences Ltd., Ireland), centrifuged, counted using a Countess™ Automated Cell Counter (Invitrogen, Bio-Sciences Ltd., Ireland) and diluted in the supplemented culture medium. The seeding concentration of A549 cells was 1.5 × 10^5^ cells/ml. MRC-5 cells were diluted at concentration of 1.5 × 10^6^ cells/ml.

#### ALI multilayered mono-cultures

Mono-cultures were grown as previously described by the authors [19] and cultured for up to 14 *d. Medium* in the basolateral chamber was changed every 3 d.

#### ALI multilayered co-cultures

Co-cultures were formed by adapting protocols previously published [21, 22]. Transwell™ supports (pore size: 0.4 μm) were inserted into the wells of 24-well plates and turned upside down. MRC-5 cells were seeded onto the basal side of the inverted inserts (final volume/support: 100 μl; cells concentration: 1.5 × 10^5^ cells/cm^2^). Plates were then closed using the bottom of the plate as lid, and incubated upside down in humidified atmosphere at 37 °C and 5% CO_2_ for 24 h, to allow cell attachment to the membrane. After 24 h, Transwell™ supports were turned in the upright position, washed with phosphate-buffered saline (PBS) and transferred into new 24-well plates where 700 μl supplemented MEM medium was previously added to the wells. A549 cells were then seeded on the apical side of the Transwell™ supports (final volume/support: 200 μl; cells concentration: 1.5 × 10^5^ cells/cm^2^). After 24 h at 37 °C and 5% CO_2_, the media in the apical compartment was removed, leaving A549 cells under ALI conditions. The ALI multilayered co-cultures were cultured for up to 14 d and medium in the basolateral chamber was changed every 3 d.

### Characterization of the in vitro models

#### Cell viability and cytotoxicity responses

A panel of commercially available assays previously reported to be suitable for screening complex 3D cultures [19, 23] were used.

##### Quantification of percentage of live A549 cells

At each time−/end-point of interest, the percentage of A549 live cells was quantitatively determined by means of BD Accuri® C6 flow cytometer (Becton Dickinson Biosciences, Oxford, UK), as previously described [19]. Measurements for each sample were carried out in duplicate to ensure data reliability. Two replicates of the same sample were included in each test (n_replicates_ = 2), and experiments repeated three times (n_tests_ = 3). Results are presented as average ± standard error of the mean. Quantitative results were confirmed by Laser Scanning Confocal Microscopy (LSCM) inspection of the live specimens stained with Hoechst 33342 and ethidium homodimer-1 (Eth-1) (Invitrogen, Bio-Sciences Ltd., Ireland) (40 min, ambient temperature).

##### Quantification of ATP levels

At the time−/end-points of interest, ATP levels were quantified by the CellTiter Glo® 3D Reagent (Promega, MyBio, Ireland), as previously described [19]. Each time−/end-point was tested in duplicate (n_replicates_ = 2), and experiments repeated three times (n_tests_ = 3). Results are presented as average ± standard error of the mean.

##### Lactate dehydrogenase (LDH) cytotoxicity assay

Supernatants were harvested at the time- and end-point under investigation, and the percentage (%) cytotoxicity was quantified by Thermo Scientific Pierce LDH Cytotoxicity Assay Kit (Fisher Scientific, Ireland), following the procedure previously described by the authors [19]. Untreated cultures and in vitro models exposed to LDH Lysis Buffer (1× in supplemented medium) for 45 min at 37 °C were included in the experimental design as negative (NT) and positive (PT) controls, respectively. Each time−/end-point was tested in duplicate (n_replicates_ = 2), and experiments were repeated three times (n_tests_ = 3). Data are presented as average ± standard error of the mean.

#### Lucifer yellow (LY) permeability assay

The crossing of LY (Sigma-Aldrich, Ireland) from the apical to the basolateral compartment of ALI multilayered co-cultures was used to investigate the confluency and integrity of the epithelial layer, as described by Dekali et al. [24]. The protocol for this assay and the methodology to extrapolate the apparent permeability coefficient (P_app_) have been previously described by the authors [19].

#### Quantification of secreted EGF and TGF-β1

Supernatants harvested from ALI multilayered mono−/co-cultures were tested by ELISAs. Supernatants were harvested from two independent experiments (n_tests_ = 2) and tested in duplicate (n_replicates_ = 2). The amount of human epidermal growth factor (EGF), total transforming growth factor beta-1 (TGF-β1), and free-active TGF-β1 were measured using the following kits: Human EGF ELISA kit (Sigma Aldrich, Ireland), LEGEND MAX™ Total TGF-β1 ELISA, and LEGEND MAX™ Free Active TGF-β1 ELISA (purchased from BioLegend, Medical Supply Co. Ltd., Ireland). Assays were carried out as per manufacturers’ protocols. Optical density of each well at λ = 450 nm was determined using an Epoch microplate reader (Biotek, Mason Technologies, Ireland), and was corrected by subtracting the optical aberration of the 96-well plastic plate at λ = 570 nm. For measuring total TGF-β1 levels, supplemented cell medium was also tested as a control, as FBS contains high levels of this growth factor. Cell medium absorbance values were then subtracted from those of samples.

#### MDR assay

The MDR Assay Kit - flow cytometry (green) (Abcam, Ireland) was used to quantitatively monitor the function and expression of three clinically important MDR transporter proteins, namely: ABCB1/MDR1, MRP1/2 and BCRP. Cell cultures were disaggregated by TryplE™ (10 min, 37 °C) and by pipetting vigorously. Cells were then resuspended in pre-warmed, phenol red-free, supplemented DMEM medium. When studying ALI multilayered co-cultures, only cells growing on the apical side of the Transwell™ supports (A549 cells) were analysed. Samples were stained according to the supplier’s protocol. Addition of specific inhibitors of the various ABC transporter proteins, included in the assay kit, allowed for the differentiation between the three pumps types and their function. Experiments were performed in triplicate for ALI multilayered mono-cultures (n_test_ = 3) and in quadruplicate for ALI multilayered co-cultures (n_test_ = 4), with three replicates of the same sample included in each experiment (n_replicates_ = 3). Data were collected by means of a BD Accuri® C6 flow cytometer (Becton Dickinson Biosciences, Oxford, UK). Mean fluorescence intensity (MFI) values and multidrug resistance activity factor (MAF) scores for each transporter were extrapolated, as for manufacturer’s instructions.

### Exposure to anti-cancer drugs

Cell cultures were exposed to four chemotherapeutic drugs: anhydrous docetaxel, vinblastine sulphate, cytarabine and methotrexate (Sigma-Aldrich, Ireland). Selection criterion was the efficacy in inducing A549 cells death based on the GDSC (Genomics of Drug Sensitivity in Cancer) database [25], as discussed in [19]. Drugs were purchased as in the form specified by the European Pharmacopoeia. In vitro models were exposed to drugs for 72 h in duplicate (n_replicates_ = 2). Experiments were repeated three times (n_tests_ = 3). One drug dose was tested for each drug. This was equal to their nominal IC_50_ concentration, as reported in the GDSC database. The efficacy of the nominal IC_50_ concentrations in inducing cell death at 72 h was validated successfully in our A549 cells batch in a previous study [19].

#### Drug solutions

In the clinic, when patients are treated by inhalation therapy, drugs are first dispersed into a hypertonic vehicle and then deposited as liquid aerosols onto the air-facing lung epithelium by means of a nebulizer. To replicate such physiological drug administration in our study, drugs were dispersed in physiological hypertonic saline (0.9% NaCl solution) supplemented with 1.25 mM CaCl_2_ and 10 mM HEPES (all purchased from Sigma-Aldrich, Ireland).

#### Exposure by direct inoculation (pipetting)

A small volume (30 μl) of drugs solution was administered by pipette to the apical side of the ALI cultures, as previously described [19, 26]. Cell cultures exposed to saline were used as negative controls (NT). This methodology mimics the layer of liquid depositing onto the air-facing lung epithelium in patients exposed to drug inhalation in the clinical settings.

#### Nebulization (Aeroneb® Pro nebuliser)

A small-volume nebulizer based on vibrating-mesh technology (Aeroneb® Pro nebuliser, Aerogen Ltd., Galway, Ireland) was used, as previously described [19].

### Cell response to drug exposure

The percentage of live A549 cells, as well the cytotoxicity, following drug exposure were quantified by flow cytometry and LDH cytotoxicity assay, as described in “Quantification of percentage of live A549 cells” and “Lactate dehydrogenase (LDH) cytotoxicity assay” sections.

#### Caspases 1–10 activity assay

The CasPASE™ Apoptosis Colorimetric Assay (G-Biosciences, VWR International, Ireland) was used to evaluate the activity of caspases 1–10 in A549 cells following drug exposure. Assay was carried out as previously described [19] and units of caspase activity were calculated.

#### Cytochrome C release from mitochondria

Levels of cytochrome C in the cell cytoplasm of A549 cells forming ALI multilayered co-cultures were quantified by Cytochrome c ELISA Kit (Invitrogen, Biosciences Ltd., Ireland), as previously described [19].

#### Inhibition of chemoresistance

ALI multilayered co-cultures were exposed to nine concentrations of docetaxel (ten-fold dilution series over a 10^8^-fold concentration range) in the absence and presence of inhibitors of various signalling pathways. The inhibitors used were: perifosine (2.5 μM) (Sigma-Aldrich, Ireland) and rapamycin (0.1, 1 and 100 μM) (Santa Cruz Biotechnology, Ireland). Inhibitors were diluted in drug-containing hypertonic saline at the desired concentration.

### Techniques

#### Laser scanning confocal microscopy (LSCM)

LSCM was used to assess F-actin organization, Ki67 protein expression, and production of surfactant-associated protein-A (SP-A) and -C (SP-C). LSCM imaging was carried out by means of a ZEISS 510 Meta confocal microscope equipped with a Zeiss Zen software (Carl Zeiss, Axiovert, Germany). Procedures and reagents have been previously described by the authors [19].

#### Trans-epithelial electrical resistance (TEER)

TEER measurements were performed on ALI multilayered co-cultures by means of an epithelial voltmeter (EVOM^2^, World Precision Instruments Inc., Hertfordshire, UK). Specific details on the experimental procedure have been previously described by the authors [19]. EVOM was preferred to other methods as it is still considered the gold-standard technique for measuring the TEER [27]. To minimise reading errors, measurements were repeated three times for each sample.

#### Cell lysis, SDS-PAGE and western immuno-blotting

Experimental conditions have been previously reported by the authors [19]. The protein content of each sample was quantified prior to analysis using the Pierce BCA Protein Assay Kit (Product no 23225; Thermo Scientific, Fisher Scientific, Ireland). Protein loadings equal to 50 μg/ml were used for all samples, to allow comparisons in protein expressions. Also, β-actin, α–tubulin or GADPH were used as protein loading controls. Primary antibodies used in this study for western blotting analysis are reported in Table 1. Relative protein expression levels were quantified by ImageJ software.

**Table 1:** Primary antibodies used for western blotting analysis in this study. Antibodies dilutions and the diluent in which they were prepared are also specified. With the exception of Anti-Surfactant protein D antibody [12G5] (Abcam, Ireland) and mouse anti-human fibronectin N-terminal monoclonal antibody (Millipore Merck, Ireland), all antibodies were purchased from Cell Signaling Technology Inc. (Brennan & Company, Ireland)

Antibody	Dilution	Diluent
BCL-2 (D55G8) Rabbit mAb (Human Specific)	1:1000	5% BSA in TBS-T 1×
Phospho-MDM2 (Ser166) Antibody	1:1000	5% BSA in TBS-T 1×
Phospho-p53 (Ser15) (16G8) Mouse mAb	1:500	5% BSA in TBS-T 1×
Akt (pan) (C67E7) Rabbit mAb	1:1000	5% BSA in TBS-T 1×
Phospho-Akt (Ser473) (D9E) XP® Rabbit mAb	1:2000	5% BSA in TBS-T 1×
Phospho-p44/42 MAPK (Erk1/2) (Thr202/Tyr204) Antibody	1:1000	5% BSA in TBS-T 1×
Mouse anti-human fibronectin N-terminal monoclonal antibody	1:1000	5% BSA in TBS-T 1×
Anti-Surfactant protein D antibody [12G5]	1:20000	5% BSA in TBS-T 1×
phospho-mTOR (Ser2448) (D9C2) XP® Rabbit mAb	1:1000	5% BSA in TBS-T 1×
c-IAP1 (D5G9) Rabbit mAb	1:1000	5% BSA in TBS-T 1×
c-IAP2 (58C7) Rabbit mAb	1:1000	5% BSA in TBS-T 1×
MCL-1 (D35A5) Rabbit mAb	1:1000	5% BSA in TBS-T 1×
MDR1/ABCB1 (D3H1Q) Rabbit mAb	1:1000	5% BSA in TBS-T 1×
Vimentin (D21H3) XP® Rabbit mAb	1:1000	5% BSA in TBS-T 1×
Phospho-SMAD2 (Ser465/467) (138D4) Rabbit mAb	1:1000	5% BSA in TBS-T 1×
β-Actin Antibody	1:1000	5% BSA in TBS-T 1×
α–Tubulin Antibody	1:1000	5% BSA in TBS-T 1×
Caspase-3 Antibody	1:1000	5% non-fat dry milk in TBS-T 1×
Cleaved Caspase-3 (Asp175) (5A1E) Rabbit mAb	1:1000	5% non-fat dry milk in TBS-T 1×
PARP (46D11) Rabbit mAb	1:1000	5% non-fat dry milk in TBS-T 1×
MRP1/ABCC1 (D708N) Rabbit mAb	1:1000	5% non-fat dry milk in TBS-T 1×
Bcl-xl (54H6) Rabbit mAb	1:1000	5% non-fat dry milk in TBS-T 1×
Survivin (6E4) Mouse mAb	1:1000	5% non-fat dry milk in TBS-T 1×
E-Cadherin (4A2) Mouse mAb	1:1000	5% non-fat dry milk in TBS-T 1×
GADPH (D16H11) XP® Rabbit mAb	1:1000	5% non-fat dry milk in TBS-T 1×

### Statistical analysis

Graph-Pad Prism (Graph-Pad Software Inc., La Jolla, CA, USA) was used to carry out the statistical analysis. A *p* value < 0.05 was considered statistically significant. The statistical tests used for each dataset are specified in the corresponding figure caption.

## Results

This study aimed at establishing an improved in vitro NSCLC model of the ALI MCC model previously developed by the authors [19]. To achieve this, an ALI multilayered co-culture of human NSCLC cells (A549 cell line) and fibroblasts (MRC-5 cells) was formed and characterized over 14-day growth. Co-culture of the two population types allowed the authors to integrate the cancer cell-fibroblast cross-talk with the 3D architecture and ALI culturing conditions of the MCC model. An in-depth study of how the cancer cell-fibroblast cross-talk affected the NSCLC cell response to four different benchmark anti-cancer drugs, delivered by direct inoculation or as a liquid aerosol by means of a clinical nebulizer, is reported in the following sections.

### Formation and characterization of ALI multilayered co-cultures over 14 days

**Fig. 1:**
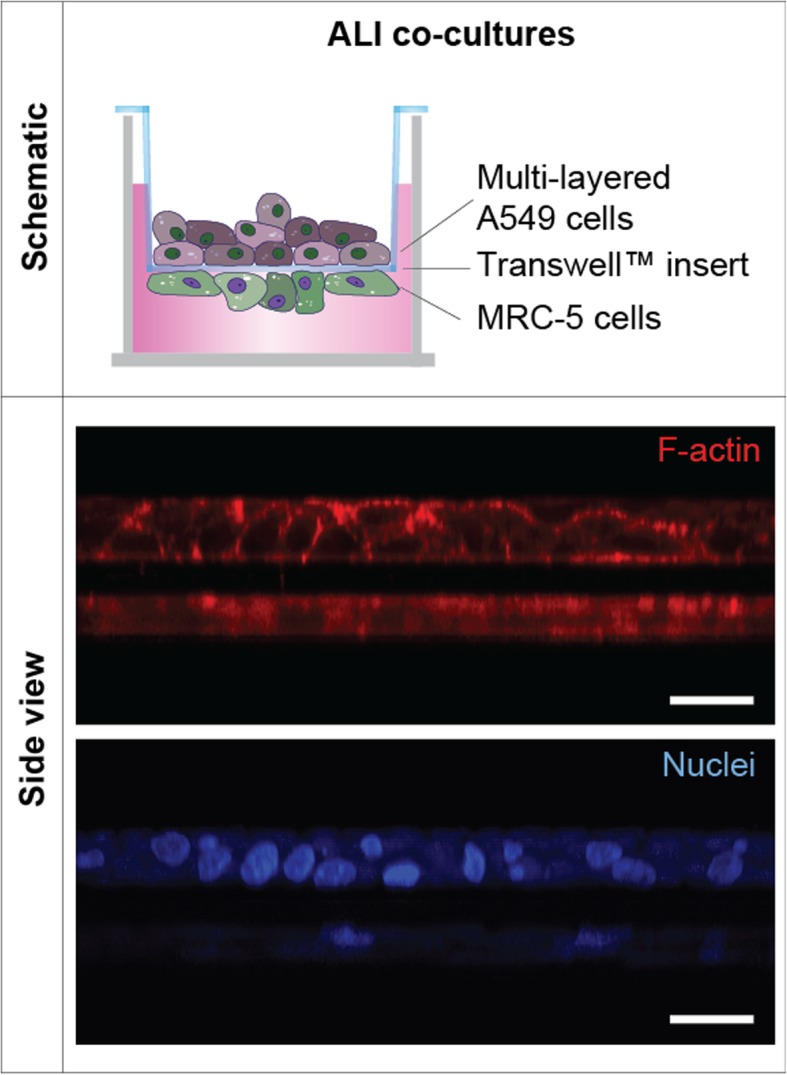
Composition and structure of ALI multilayered co-cultures. Schematics of the in vitro model developed and representative LSCM images of the culture showing the F-actin (in red) and cell nuclei (in blue) organization in these cultures. The Z-stack LSCM images, clearly demonstrating the 3D architecture of the models developed, were reconstructed with ImageJ software to obtain the side view shown. Scale bars: 20 μm (objective lens, 63×)

A549 cells successfully formed 3D multilayers when cultured together with human fibroblasts (MRC-5 cells) under ALI conditions (Fig. 1).

**Fig. 2:**
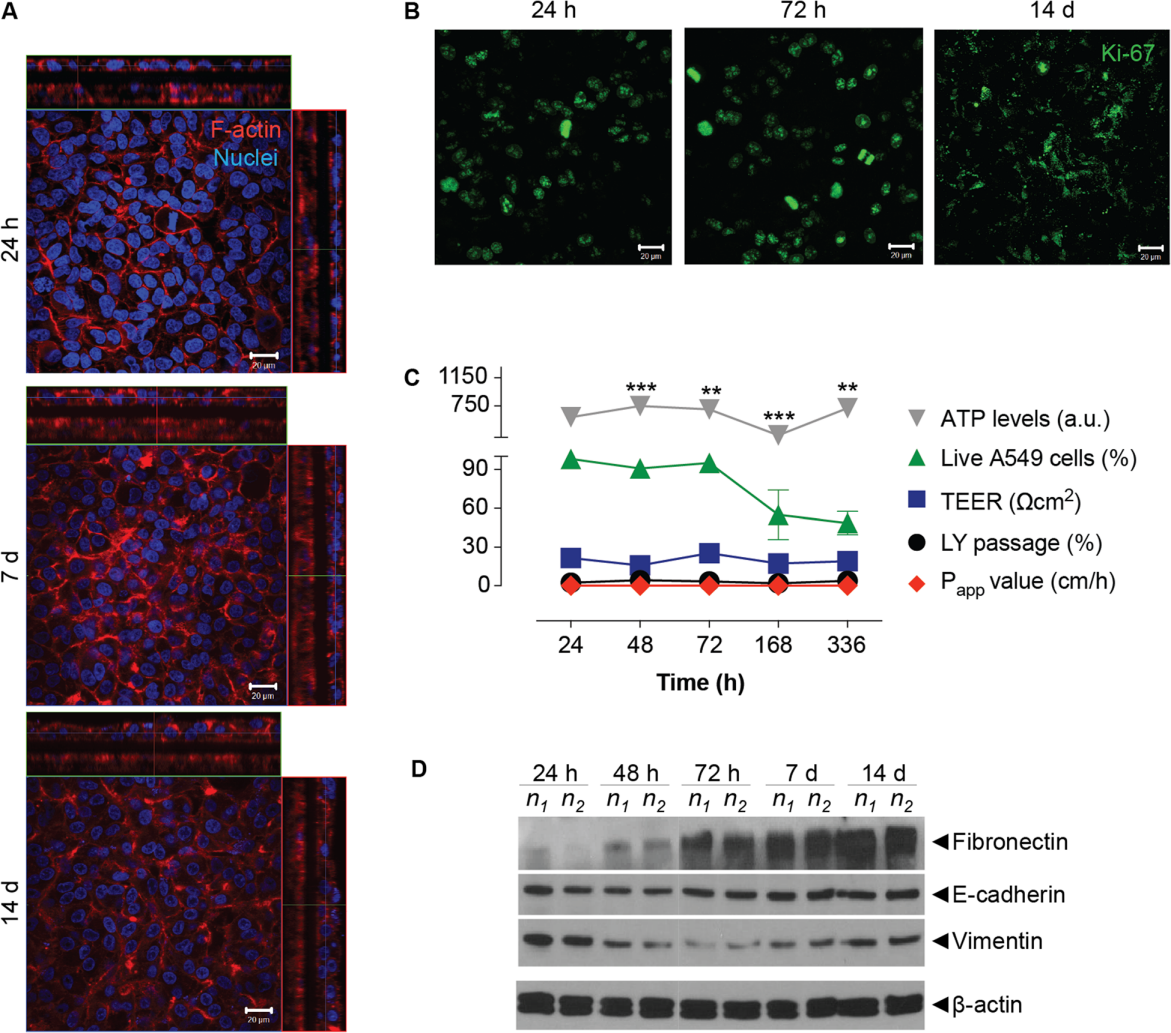
Changes in the properties of ALI multilayered co-cultures overtime. **a** and **b** Representative LSCM images of the **a** F-actin organization (in red) and **b** Ki67 protein expression (in green) in ALI multilayered co-cultures over 14-day growth. Full datasets for all time-points are reported in the Additional file 1. Scale bars: 20 μm (objective lens: 63×). **a** Cell nuclei were also stained with Hoechst 33342 (in blue). Z-stack images, here presented in orthogonal view, clearly demonstrate the multilayered structure of the in vitro models developed. **b** Z-stack images of the apical side of the cultures were reconstructed and are shown as three-dimensional projections. **c** Time-dependent changes in: ATP levels, percentage (%) of live A549 cells, TEER, % of LY passage and P_ap*p*_ values in ALI multilayered co-cultures grown up to 14 d. Data are shown as average *±* standard error of the mean (n_replicates_ = 2; n_tests_ = 3). The symbols (**) and (***) indicate statistically significant changes as compared to the values measured at 24 h (*p <* 0.01 and 0.001, respectively) (two-way ANOVA and Bonferroni post-test). **d** Western blot analysis of E-cadherin (epithelial marker), vimentin and fibronectin (mesenchymal markers) in A549 cells forming ALI multilayered co-cultures and cultured up to 14 d. The time-points examined were: 24 h, 48 h, 72 h, 7 d and 14 d. Abbreviations “*n*_1_” and “*n*_2_” indicate different experimental replicates. *β*-actin expression is also reported as protein loading control

The adenocarcinoma cell line showed a cortical organization of the F-actin (Fig. 1 and Additional file 1: Video S1). Such organization did not modify overtime (Fig. 2a and Additional file 2: Figure S1). MRC-5 cells density also increased overtime, forming a 2–3 layers thick tissue after 14 d in culture (Fig. 2a). Such tissue was constituted by a complex network of cells with fibers oriented in various directions (Additional file 1: Video S1 and Additional file 2: Figure S1).

Proliferative activity (here quantified as Ki67 protein expression) was detected in A549 cells over 14 d in culture, with Ki67-positive cells found in all layers and observed throughout the ALI multilayered co-cultures (Fig. 2b and Additional file 2: Figure S1). Ki67 protein was found in the A549 cell nuclei over 72 h of culture (Additional file 2: Table S1). In contrast, it localized within compartments of the cell body from 7 d onwards (Additional file 2: Table S1). With regards to MRC-5 cells, the Ki67 protein expression was low at all time-points (Additional file 2: Figure S1). This was probably due to the longer doubling time of this cell line as compared to A549 cells, as well as to its non-malignant nature.

A decrease in the percentage (%) of live A549 cells was evidenced in ALI multilayered co-cultures at 7 d, although such decrease was not statistically significant (Fig. 2c). Time-dependent changes in the viability of A549 cells in ALI multilayered co-cultures were also confirmed by the qualitative microscopy imaging (Additional file 2: Figure S2). Nevertheless, ATP levels were constant in ALI multilayered co-cultures over 14 d (Fig. 2c).

ALI multilayered co-cultures were not permeable to Lucifer Yellow (LY), a barrier integrity marker, at all time-points, showing P_app_ values and LY passage equal or close to zero (Fig. 2c). Nevertheless, the reduced culture permeability did not confer any measurable Trans-Epithelial Electrical Resistance (TEER) (Fig. 2c), one of the key features defining the formation of an epithelial barrier.

Next, we investigated the expression of Epithelial-to-Mesenchymal (EMT) protein markers in ALI multilayered co-cultures by western blot techniques. Both E-cadherin (epithelial marker) and the mesenchymal protein vimentin were expressed at all time-points tested (Fig. 2d). Fibronectin expression (mesenchymal marker) was absent at 24 h but was detected after 48 h growth.

### ALI multilayered co-cultures are chemoresistant

**Fig. 3:**
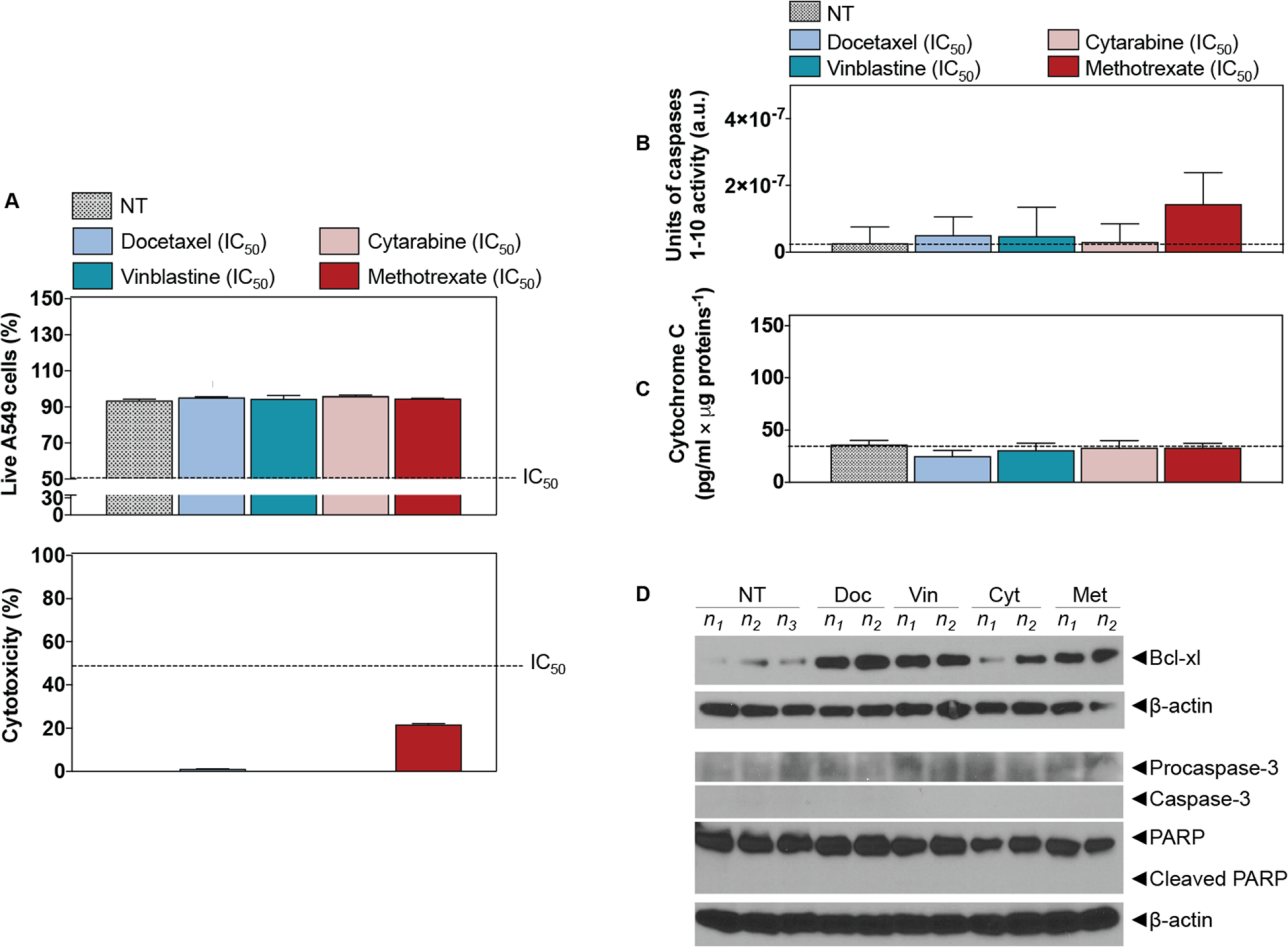
Chemoresistance evaluation in ALI multilayered co-cultures exposed to anti-cancer drugs by direct inoculation. **a** Percentage (%) of (from top to bottom) live A549 cells and cytotoxicity (detected by LDH cytotoxicity assay) in ALI multilayered co-cultures grown for 72 h and exposed to four different anti-cancer drugs (docetaxel, vinblastine, cytarabine and methotrexate) for 72 h. Drugs were tested at their nominal IC_50_ concentration. Data are reported as average *±* standard error of the mean (n_replicates_ = 2; n_tests_ = 3). No significant difference from the untreated cultures (NT) (one-way ANOVA with Dunnett post-test). **b** and **c** Histograms showing the **b** units of caspases 1–10 activity and **c** the levels of cytochrome c released from the mitochondria into the cell cytoplasm as detected in ALI multilayered co-cultures grown for 72 h and then exposed to four anti-cancer drugs at their nominal IC_50_ for 72 h. Untreated cultures were also tested as negative control (NT). Dotted lines indicate the levels of caspases activity and cytochrome c release in NT. Data are presented as average *±* standard error of the mean (n_replicates_ = 2; n_tests_ = 3). No significant differences from NT were found (one-way ANOVA with Dunnett post-test). **d** Western blot analysis of the expression of Bcl-xl, procaspase-3 and caspase-3, PARP and its cleaved form (cleaved PARP) in A549 cells forming ALI multilayered mono-cultures. Cultures were grown for 72 h and then exposed to docetaxel (Doc), vinblastine(Vin), cytarabine (Cyt) or methotrexate (Met) at their nominal IC_50_ for 72 h. Abbreviations “*n*_1_”, “*n*_2_” and “*n*_3_” indicate different biological replicates. *β*-actin expression is also reported as protein loading control

ALI multilayered co-cultures were exposed to four chemotherapeutic agents (namely, docetaxel, cytarabine, vinblastine and methotrexate) at their nominal IC_50_ concentration by direct inoculation. ALI multilayered co-cultures showed a small or nil degree of response to drug treatments (Fig. 3a). Furthermore, the absence of cell death/cytotoxicity in ALI multilayered co-cultures exposed to the four anti-cancer agents was correlated to a complete lack in: (i) caspases 1–10 activation (Fig. 3b), (ii) release of cytochrome c (Fig. 3c), and (iii) subsequent activation of procaspase-3 and PARP into their active cleaved forms (Fig. 3d). Expression of Bcl-xl, an anti-apoptotic protein, was increased in drug-treated cultures (Fig. 3d).

### ALI multilayered co-cultures are more chemoresistant than mono-cultures

The viability of ALI multilayered co-cultures following drug exposure was compared to that of ALI multilayered mono-cultures exposed to the same treatments. Drugs were administered by direct inoculation or via nebulization.

**Fig. 4:**
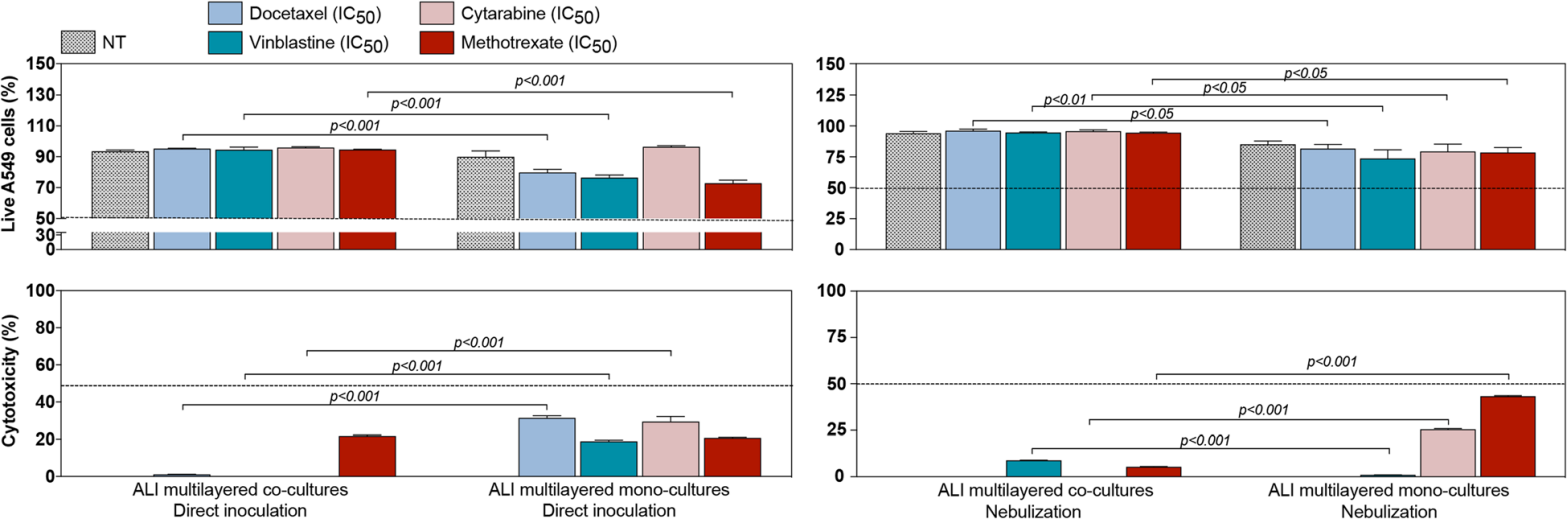
Comparison of the chemoresistance of ALI multilayered co- and mono-cultures. Percentage (%) of live A549 cells and cytotoxicity detected in ALI multilayered mono- and co-cultures exposed to four anti-cancer drugs (docetaxel, cytarabine, vinblastine and methotrexate) at their nominal IC_50_ concentration for 72 h, by direct inoculation or nebulization. Data are reported as average *±* standard error of the mean (n_replicates_ = 2; n_tests_ = 3). *p* values indicate significant differences (two-way ANOVA and Bonferroni post-test)

Significant differences were observed between the responses of the two in vitro models, with ALI multilayered co-cultures showing a small or nil degree of response to inoculated drug treatments (Fig. 4), with negligible cytotoxicity and an increased percentage of live A549 cells as compared to ALI multilayered mono-cultures (Fig. 4 and Additional file 2: Figure S3). Interestingly, ALI multilayered co-cultures generally showed an increased chemoresistance compared to mono-cultures even when drugs were nebulized (Fig. 4). With the exception of vinblastine, in fact, the cytotoxic effect of anti-cancer drugs administered as a liquid aerosol by nebulization significantly decreased in ALI multilayered co-cultures, as compared to drugs administered by direct inoculation (Additional file 2: Figure S4). A similar trend was previously reported for ALI multilayered mono-cultures [19].

**Fig. 5:**
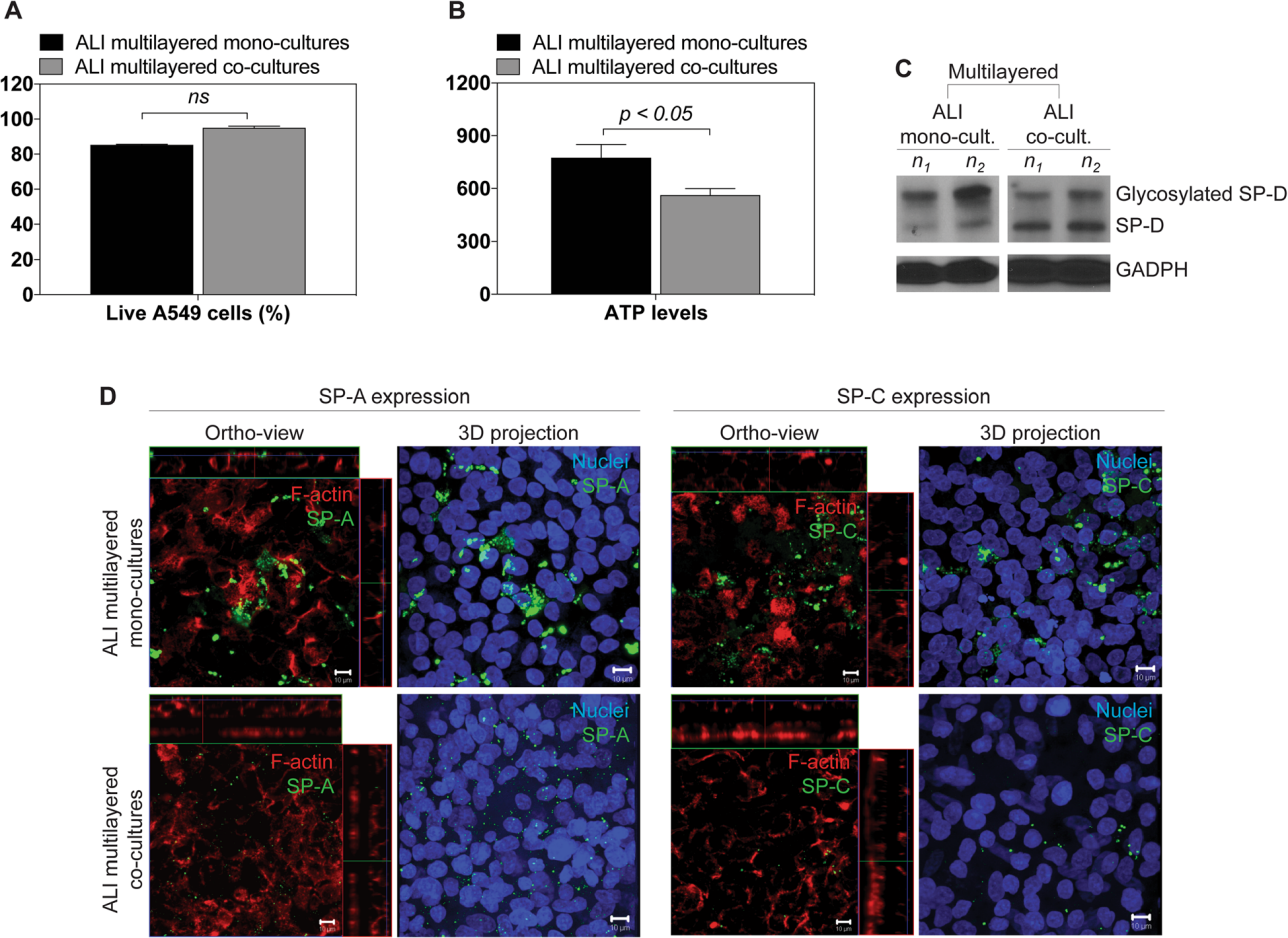
Differences in the functional and morphological properties of ALI multilayered mono- and co-cultures. **a** and **b** Percentage (%) of **a** live A549 cells and **b** total ATP levels detected in ALI multilayered mono- or co-cultures. Significant differences were detected among the ATP levels of the two cultures (one-way ANOVA with Dunnett post-test). **c** Western blotting analysis of the expression of surfactant-associated protein-D (SP-D) in ALI multilayered mono- or co-cultures. Both SP-D monomeric subunit were detected: the dominant form at 40 kDa and the additional subunit at 50 kDa (glycosylated SP-D). Abbreviations “*n*_1_” and “*n*_2_” indicate different biological replicates. GADPH expression is also reported as protein loading control. **d** Representative LSCM images of (from top to bottom) ALI multilayered mono- and co-cultures. ALI MCCs were stained for (from left to right) surfactant-associated protein-A (SP-A) and -C (SP-C) (both in green). The uneven staining was probably associated to the poor ability of A549 cells to produce surfactant-associated proteins. Cells were also stained for F-actin (in red) and nuclei (in blue). Z-stack images of the cultures were reconstructed and are shown in orthogonal view (ortho-view) or as three-dimensional projections. Scale bars: 10 μm (objective lens: 63×)

It should be noted that, untreated ALI multilayered co-cultures grown for 72 h and used within this study exhibited a cell viability comparable to that of mono-cultures grown for 14 days, as measured based on the percentage of live A549 cells (Fig. 5a). However, a significant difference in the total ATP content was found, with ALI multilayered co-cultures showing the lower levels (Fig. 5b). While surfactant-associated proteins D (SP-D) expression was similar between mono- and co-cultures (Fig. 5c), surfactant-associated proteins A (SP-A) and C (SP-C) were observed predominantly in ALI multilayered mono-cultures (Fig. 5d). Interestingly, two distinct bands could be detected in the A549 cells’ lysates when probing the SP-D protein, in both cultures (Fig. 5c). These bands corresponded to the dominant form of human SP-D monomers with a molecular mass of around 40 kDa [28, 29], and a variant form of monomeric subunit, with a molecular mass equal to 50 kDa [30]. The increased molecular weight of the variant form is due to *O*-linked glycosylation of the SP-D protein [30].

### In ALI multilayered co-cultures, MultiDrug Resistance (MDR) is triggered by cancer cell-fibroblast cross-talk

Following drug exposure, ALI multilayered co-cultures showed a decrease in the expression of the MRP1/ABCC1 drug efflux pump that was shown to drive chemoresistance in ALI multilayered mono-cultures [19] (Additional file 2: Figure S5). Nevertheless, functional detection and profiling of multidrug resistant A549 cell phenotypes in live ALI MCCs demonstrated that, MDR was triggered by MRP1/2 transporters in both ALI multilayered mono- and co-cultures (Additional file 2: Figure S6), with MAF (multidrug resistance activity factor) scores of around 40 for this drug efflux pump. It should be noted that MAF values above 25 are considered indicative of MDR-positive specimens.

Based on the results showing that ALI multilayered co-cultures were more chemoresistant than mono-cultures (Fig. 4), we hypothesized that a cancer cell-fibroblast cross-talk was established within the ALI multilayered co-cultures, thus inducing further MDR. Here we investigated if two specific stimuli, implied in such cross-talk in in vivo conditions, could be detected in our co-culture model: (i) direct cell-to-cell contact and (ii) secretion of biochemical mediators (e.g. growth factors, cytokines) [31].

**Fig. 6:**
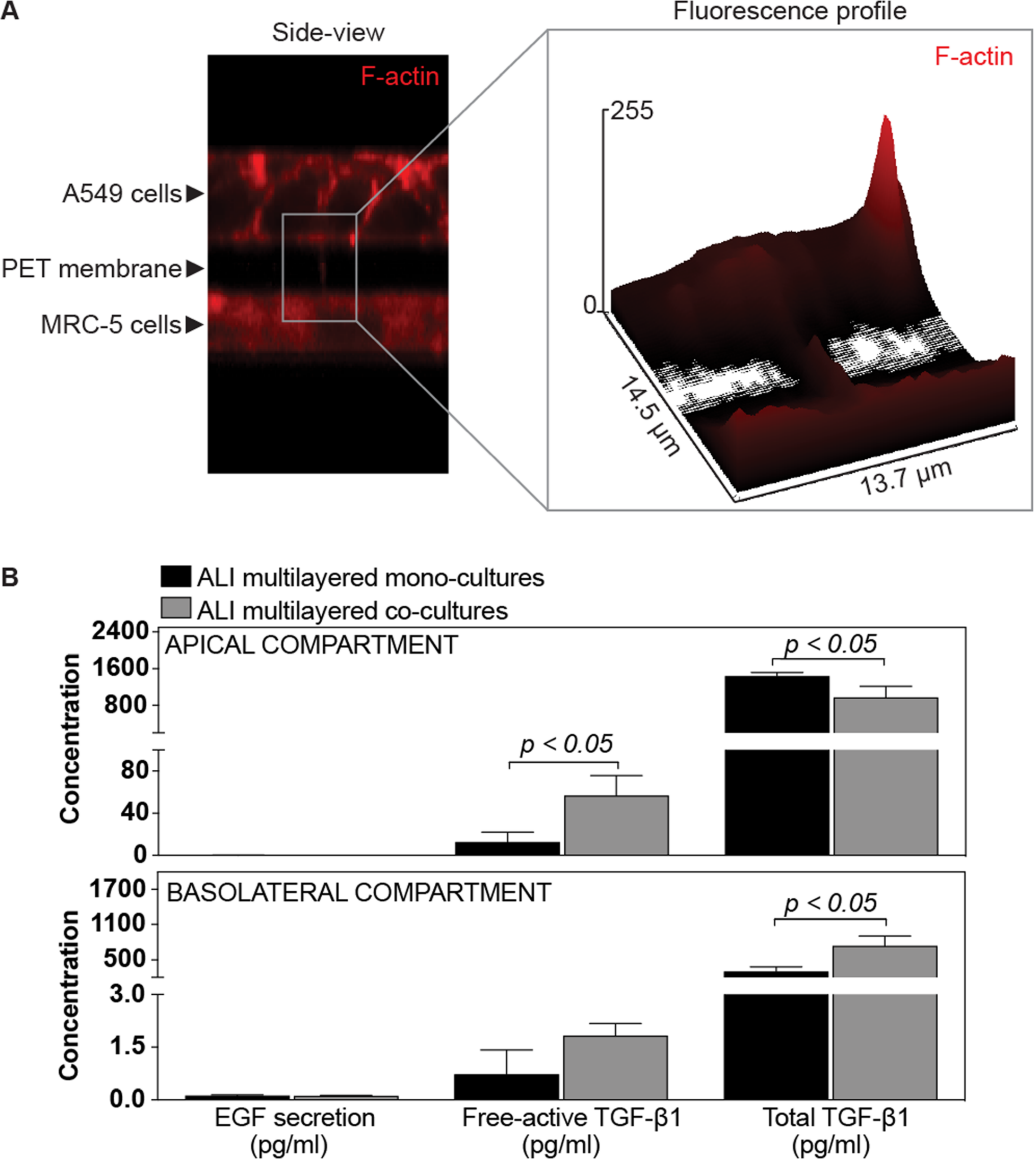
Investigation of cancer cell-fibroblast cross-talk through physical and chemical stimuli. **a** Representative LSCM image of ALI multilayered co-cultures. Cells were stained for F-actin (in red) and Z-stack images of the cultures were reconstructed and are shown in side view mode. The right hand-side of (**a**) shows the red fluorescence intensity profile of the selected region of interest from **a** (left hand-side). An F-actin filament extending from the apical to the basolateral side is visible. Objective lens: 63×. **b** Concentration of EGF, free-active TGF-*β*1 and total TGF-*β*1 detected in the supernatants harvested from the apical (top graph) and basolateral (bottom graph) compartments of the Transwell*™* supports when A549 cells were cultured as ALI multilayered mono- or co-cultures. *p* values < 0.05 indicate significant differences (one-way ANOVA followed by Dunnett post-test)

Direct cancer cell-fibroblast contact was impaired in our co-culture model, as the 10 μm-thick PET membrane of the Transwell™ inserts physically separated the A549 cells (growing on the apical side) from the MRC-5 cells (submerged in the basolateral compartment) (Fig. 1). Interestingly, we found that, in ALI multilayered co-cultures, few F-actin filaments extended through the PET membrane pores, between A549 and MRC-5 cells (Fig. 6a). Similar structures could not be identified in ALI multilayered mono-cultures (data not shown). The presence of such structures could suggest that a partial cell-to-cell contact was established within ALI multilayered co-cultures. To allow any conclusion, further studies would require the identification of the F-actin structures detected in ALI multilayered co-cultures as intercellular bridges for cellular communication [32] or simply membrane protrusions of invading A549 cells.

The secretion of two biochemical mediators, the epidermal growth factor (EGF) and transforming growth factor-β (TGF-β), was studied and their levels quantified in the supernatants harvested from both the apical and basolateral compartments of the ALI multilayered co-cultures (Fig. 6b). Secretion levels were compared to those found for ALI multilayered mono-cultures as benchmark. It should be noted here that, in our study, we monitored the expression levels only of the TGF-β1 isoform, as TGF-β1 upregulation is the most frequent in lung cancer [33]. Studies on NSCLC patients’ tissue specimens have also shown that TGF-β1 promotes tumor progression [34, 35].

No significant EGF secretion could be detected in either ALI multilayered mono- or co-cultures (Fig. 6b). In contrast, significant amounts of free-active TGF-β1, the ligand that binds the TGF-β receptor (TGF-βR) and exerts the signaling functions, were detected in both MCCs (Fig. 6b). This was found to be consistent with previous reports [36]. The levels of free-active TGF-β1 secreted by ALI multilayered co-cultures were always higher than those detected in mono-cultures (Fig. 6b).

### A549 cells cultured as ALI multilayered co-cultures show activation of the PI3K/AKT signaling cascade

**Fig. 7:**
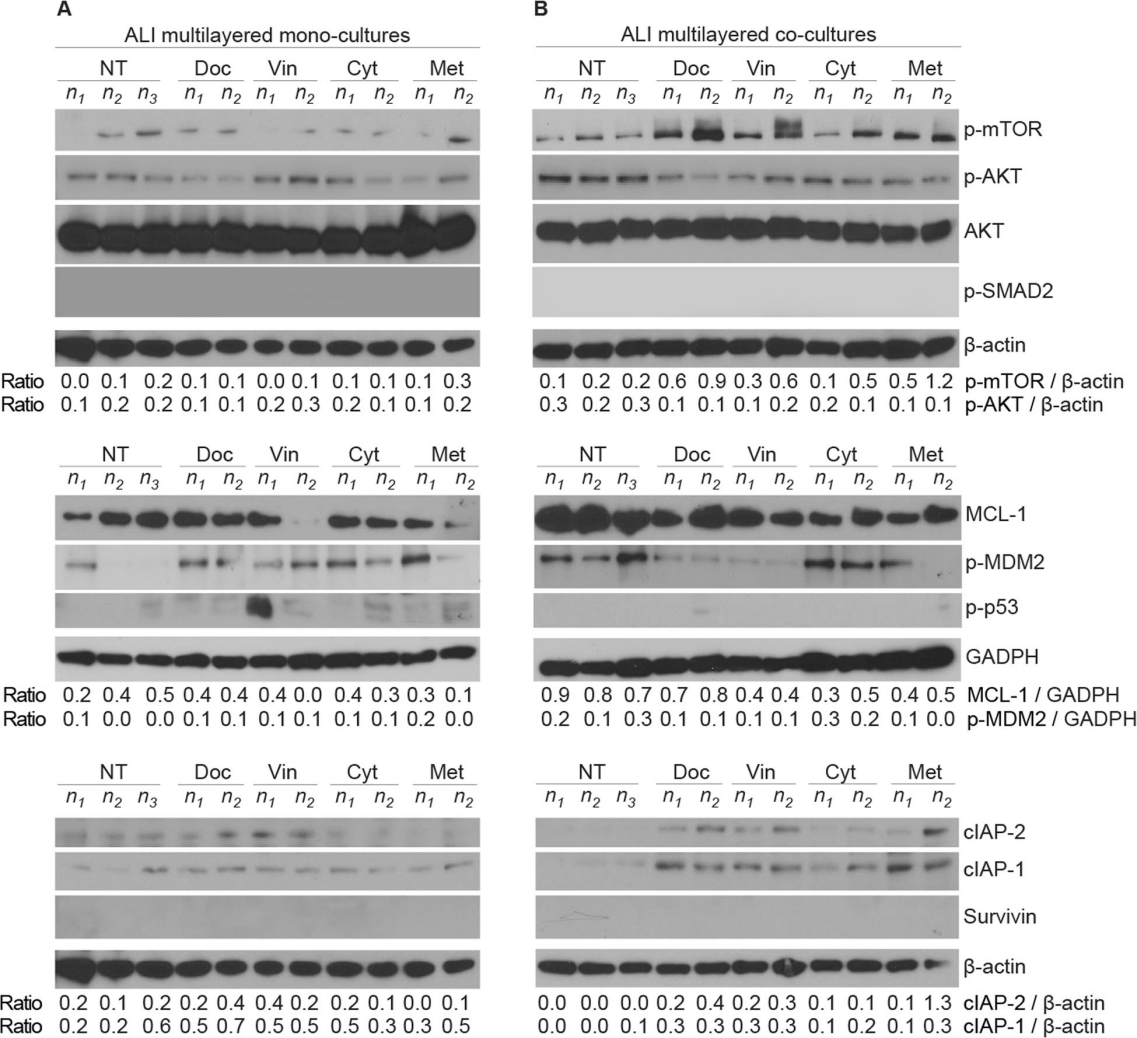
Activation of MDR signalling cascades in ALI multilayered cultures exposed to anti-cancer drugs by direct inoculation. Western blot analysis of the expression of various proteins in A549 cells forming **a** ALI multilayered mono-cultures or **b** ALI multilayered co-cultures. Proteins analysed are: phospho-mTOR (p-mTOR), phospho-AKT (p-AKT), AKT, phospho-SMAD2 (p-SMAD2), MCL-1, phospho-MDM2 (p-MDM2), phospho-p53 (p-p53), cIAP-1, cIAP-2 and survivin. Cultures were exposed to docetaxel (Doc), vinblastine (Vin), cytarabine (Cyt) or methotrexate (Met) at their nominal IC_50_ concentration for 72 h. The expression of the proteins under study in untreated cultures (NT) is also reported for comparison. Abbreviations “*n*_1_”, “*n*_2_” and “*n*_3_” indicate different biological replicates. β-actin and GADPH expressions are reported as protein loading control

Although our cell line batch demonstrated to be capable to activate the SMAD2 signalling pathway when stimulated with human recombinant TGF-β1 (Additional file 2: Figure S7), in agreement with the literature, no phospho-SMAD2 (p-SMAD2) could be detected in A549 cells lysates of untreated (NT) or drug-treated ALI multilayered co-cultures (Fig. 7).

TGF-β1 can also activate protein kinase B (known as AKT) through TGF-βR-induced phosphatidylinositol-3-kinases (PI3K), which phosphorylates AKT (Additional file 2: Figure S8). Phosphorylated AKT (p-AKT) was detected in ALI multilayered co-cultures (Fig. 7b). Its expression in untreated ALI multilayered co-cultures was slightly higher than that detected in mono-cultures (Fig. 7a). Following exposure to the anti-cancer drugs for 72 h it remained almost unchanged (Fig. 7b).

**Fig. 8:**
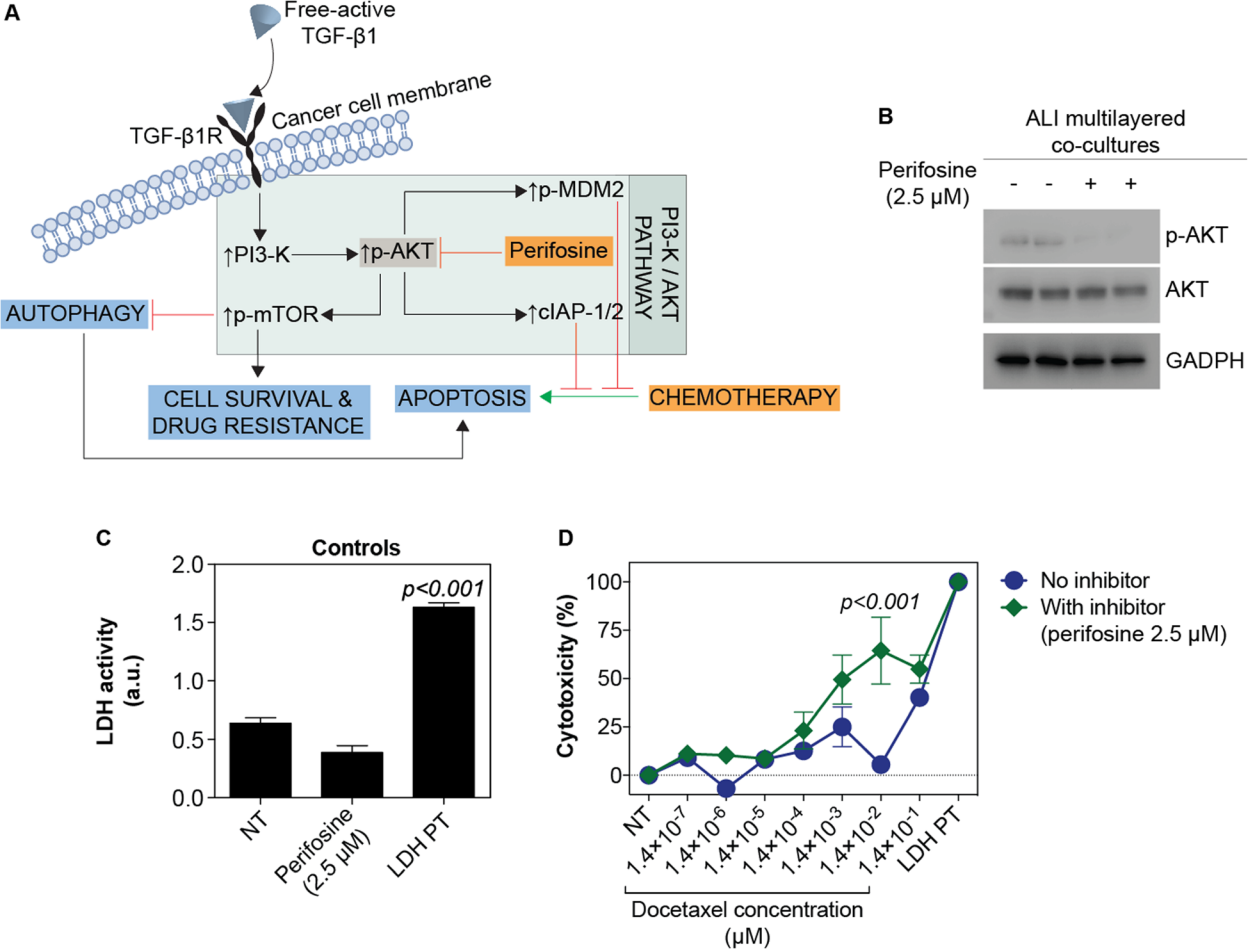
Drug response of ALI multilayered co-cultures in the presence of perifosine, an inhibitor of phospho-AKT. **a** Simplified schematics of the PI3K/AKT/mTOR signalling cascade and perifosine inhibitory effect. **b** Western blot analysis of the expression of AKT and phospho-AKT (p-AKT) in ALI multilayered co-cultures grown for 72 h and exposed to perifosine (2.5 μM; 72 h) by direct inoculation. Untreated cultures (−) were also analysed as controls. GADPH expression was the protein loading control. **c** Histogram of the LDH activity in the experimental controls: untreated ALI multilayered co-cultures (NT), ALI multilayered co-cultures exposed by direct inoculation to perifosine (2.5 μM) for 72 h, and positive control (LDH PT). No significant increase in LDH activity was detected following perifosine treatment as compared to NT. Data are reported as average *±* standard error of the mean (n_replicates_ = 2; n_tests_ = 3). *p <* 0.001 indicates a significant difference from NT (one-way ANOVA with Dunnett post-test). **d** Percentage (%) cytotoxicity detected by LDH cytotoxicity assay in ALI multilayered co-cultures grown for 72 h and exposed to 10 concentrations of docetaxel for 72 h, in the presence or absence of perifosine (2.5 μM). Cell cultures were exposed to drugs by direct inoculation. Values for untreated cultures (NT) and positive control (LDH PT) are also shown. Data are reported as average *±* standard error of the mean (n_replicates_ = 2; n_tests_ = 3). *p <* 0.001 indicates a significant difference from NT (two-way ANOVA with Bonferroni post-test)

When inhibiting the phosphorylation of AKT with perifosine [37] (Fig. 8a-b), docetaxel efficacy was increased at most doses tested, although the increased drug cytotoxic effect was statistically significant only at one dose (1.4 × 10^− 2^ μM) (Fig. 8d). No significant increase in LDH activity was induced by perifosine (concentration of 2.5 μM), when ALI multilayered co-cultures were exposed to it in the absence of docetaxel (Fig. 8c). This was consistent with previously published works [38].

It is known that the phosphorylated form of AKT can promote MDR by: (i) stimulating induced myeloid leukemia cell differentiation protein (MCL-1) that promotes EMT in lung cancer cells [39]; (ii) triggering phosphorylation of anti-apoptotic mouse double minute 2 homolog (MDM2), which inhibits activation of the intrinsic apoptotic pathway; (iii) inducing the phosphorylation of oncogenic mTOR, responsible for autophagy inhibition; and/or (iv) increasing the expression levels of cellular inhibitor of apoptosis protein-1 and -2 (cIAP-1/2) through nuclear factor NF-κB. This is summarised in Additional file 2: Figure S8. Our western blotting analysis showed that, the expression of MCL-1 and phosphorylated MDM2 (p-MDM2) were ubiquitously expressed in both untreated and drug-treated ALI multilayered co-cultures (Fig. 7b). The MCL-1 expression levels in ALI multilayered co-cultures were significantly higher than in mono-cultures, in both untreated and drug-treated in vitro models (Fig. 7). In contrast, phosphorylated mTOR (p-mTOR) and cIAP-1/2 proteins were up-regulated in drug-treated ALI multilayered co-cultures, suggesting an increase in some MDR features following exposure to anti-cancer drugs. Notably, in untreated cultures, the levels of p-mTOR expressed by A549 cells were comparable between ALI multilayered co-cultures and mono-cultures (Fig. 7 and Additional file 2: Figure S9). On the other hand, once exposed to the four anti-cancer drugs, the A549 cells forming ALI multilayered co-cultures showed a clear increase in the p-mTOR expression levels as compared to ALI multilayered mono-cultures exposed to the same drugs (Fig. 7 and Additional file 2: Figure S9). It is widely reported in the scientific literature that 3D cell cultures are characterized by higher variability than their 2D counterparts [40, 41]. Even considering the variability found among our biological replicates, differences in the p-mTOR expression levels were statistically significant when comparing mono- and co-cultures exposed to docetaxel and methotrexate (Additional file 2: Figure S9). Also, our data from the western blot analysis strikingly pointed out that phosphorylated p53 (p-p53), a marker of apoptosis, was completely depleted from A549 cells forming ALI multilayered co-cultures (Fig. 7b), while low levels of p-p53 could be detected in drug-treated ALI multilayered mono-cultures (Fig. 7a). The complete depletion of p-p53 could be associated to the AKT-triggered phosphorylation of MDM2, which was detected in the lysates of A549 cells isolated from ALI multilayered co-cultures (Fig. 7b).

**Fig. 9:**
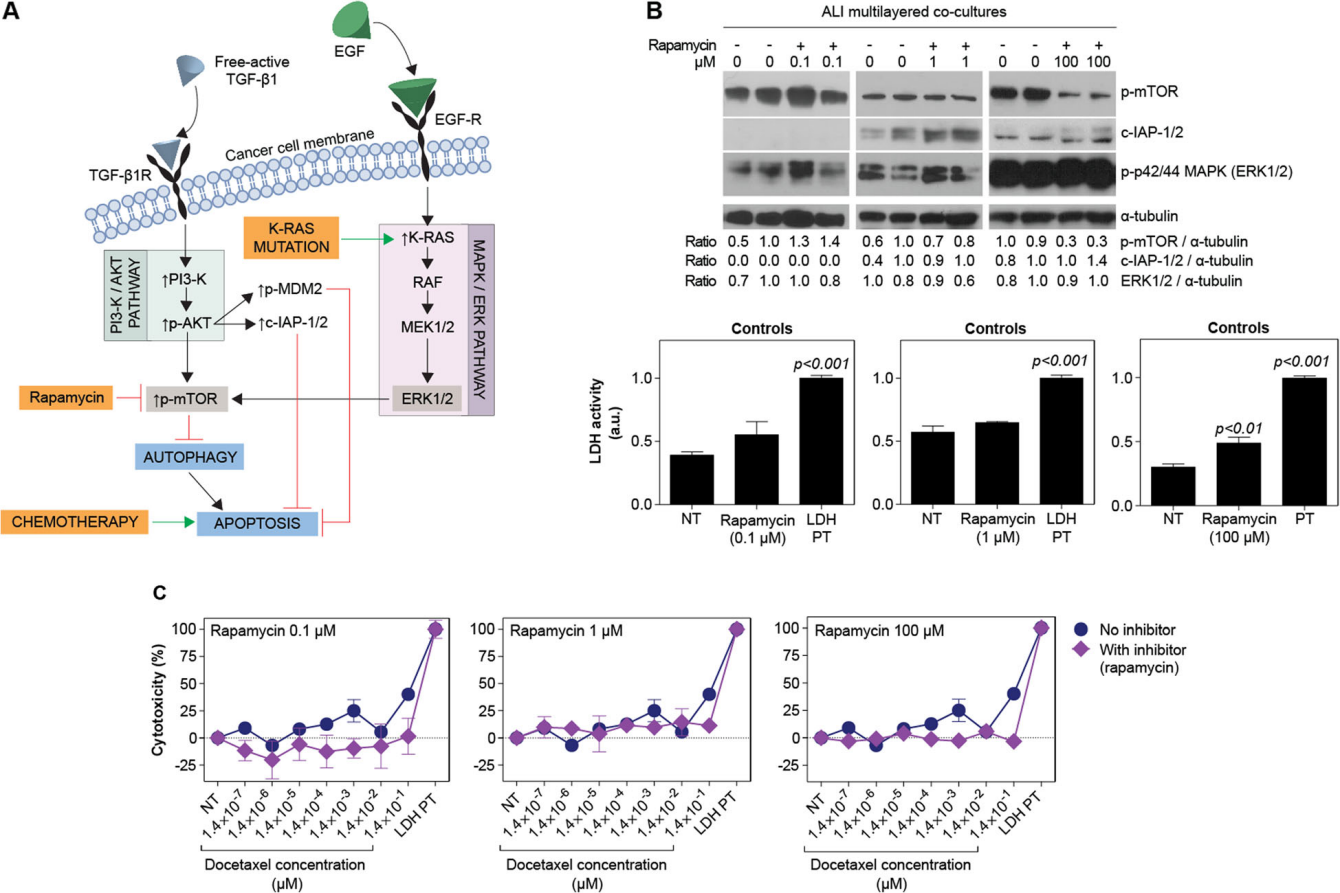
Drug response of ALI multilayered co-cultures in the presence of mTOR inhibitor rapamycin. **a** Brief schematics of the PI3K/AKT/mTOR and MEK/ERK signalling cascades and the inhibitory effect of rapamycin. **b** Western blot analysis of the expression of phospho-mTOR (p-mTOR), cIAP-1/2 and phospho-p42/44 (ERK1/2) (p-p42/44) in ALI multilayered co-cultures grown for 72 h and exposed to rapamycin (72 h) by direct inoculation. Three concentrations of rapamycin were tested: 0.1, 1 and 100 μM. Untreated cultures (−) were also analysed for comparison. *α*-tubulin expression is reported as protein loading control. Histogram of the LDH activity in the experimental controls are also reported: untreated ALI multilayered co-cultures (NT), ALI multilayered co-cultures exposed by direct inoculation to rapamycin (0.1, 1 and 100 μM) for 72 h, and positive control (LDH PT). Significant LDH activity was detected following 100 μM rapamycin treatment. Data are reported as average *±* standard error of the mean (n_replicates_ = 2; n_tests_ = 3). *p <* 0.01 and *p <* 0.001 indicate a significant difference from NT (one-way ANOVA with Dunnett post-test). **c** Percentage (%) cytotoxicity detected by LDH cytotoxicity assay in ALI multilayered co-cultures grown for 72 h and exposed to 10 concentrations of docetaxel for 72 h, in the presence or absence of rapamycin (0.1, 1 and 100 μM). Cell cultures were exposed by direct inoculation. Values for untreated cultures (NT) and positive control (LDH PT) are also shown. Data are reported as average *±* standard error of the mean (n_replicates_ = 2; n_tests_ = 3). No significant differences from NT were found (two-way ANOVA with Bonferroni post-test)

When inhibiting the phosphorylation of mTOR by rapamycin exposure (Fig. 9a-b), no increase in docetaxel cytotoxicity was detected (Fig. 9c). On the other hand, in the presence of rapamycin the expression of cIAP-1/2 increased in drug-treated ALI multilayered co-cultures, as compared to the untreated models (Fig. 9b). This was observed for rapamycin concentrations equal to 1 and 100 μM (Fig. 9b). Albeit in the literature rapamycin has been shown to induce inhibition of mTOR phosphorylation at concentration equal to 0.1 μM [42], in our experiments only partial p-mTOR down-regulation was detected at 100 μM, a concentration at which a small increase in the p-p44/42 (ERK1/2) proteins expression levels was also found (Fig. 9b). This was consistent with previous studies, showing that mTOR inhibition induces activation of the MEK/ERK signalling pathway in A549 cells [43]. Also, this is believed to be among the causes responsible for the modest clinical efficacy shown by mTOR inhibitors in cancer treatments [44–46]. Higher rapamycin doses could not be tested, since a small increase in LDH activity was found at 100 μM in ALI multilayered co-cultures thus indicating that the inhibitor triggered a small cytotoxicity response (Fig. 9b).

Finally, expression of survivin, a protein of the inhibitor of apoptosis (IAP) family, was not found in lysates of A549 cells forming ALI multilayered co-cultures (Fig. 7b).

## Discussion

For the efficacy testing of inhaled anti-cancer drugs, it is important to mimic the direct contact of the lung epithelium with the gas phase (air). ALI cultures is the only in vitro model that can reproduce this feature. The authors have previously demonstrated the advantages of using ALI multilayered mono-cultures for testing the efficacy of anti-cancer drugs delivered as liquid aerosol [19]. ALI multilayered mono-cultures incorporate, in fact, both the ALI culturing conditions and the 3D architecture of the tumour tissue, which is generally mimicked in in vitro cancer research experiments through the adoption of 3D tumour spheroids. In the present study, we report the formation and characterization of an improved in vitro ALI MCC, which has the additional advantage of being able to reproduce the cancer cell-fibroblast cross-talk and, subsequently, some of the complex networks and mechanisms triggering MDR in NSCLC patients.

Fibroblasts are one of the main cell types constituting the tumor stroma. It has been demonstrated that cancer-associated fibroblasts (CAFs) play a key role in the development of cancer cells’ adaptive resistance to chemotherapy [20], particularly in NSCLC [47, 48]. For example, CAFs have been demonstrated to drive chemoresistance to gefitinib in epithelial growth factor receptor (EGFR)-mutant NSCLC cells [49, 50]. Similarly, the efficacy of various chemotherapeutic agents (including gefitinib, docetaxel, selumetinib and trametinib) is inhibited in KRAS-mutated NSCLC cells (such as the A549 cell line) as a consequence of CAFs presence in culture [51, 52]. In the past years, it has also been shown that, upon co-culturing with CAFs, the invasive potential of the A549 cell line increases [53, 54]. Furthermore, in a recent study, human lung CAFs were shown to significantly promote the radioresistance of A549 cells [55]. Thus, we incorporated lung fibroblasts (MRC-5 cells) in the in vitro model with the aim to provide our ALI MCC with added value for drug screening applications. Our results clearly demonstrated that indeed the MDR mechanisms mimicked in vitro are strongly influenced by the presence of fibroblasts within the culture. To support our statement, an in-depth comparison with data previously published on ALI multilayered mono-cultures [19] was carried out.

LSCM images showed that co-culture of A549 cells with fibroblasts did not cause any F-actin rearrangement within lung cancer cells (Fig. 2a), as compared to ALI multilayered mono-cultures, as shown in [19]. In contrast, Ki67 cytoplasmic translocation was evidenced in ALI multilayered co-cultures (Fig. 2b). Immunohistochemistry studies have previously described membranous and cytoplasmic Ki67 protein expression in clinical samples of invasive breast carcinoma [56]. Translocation of Ki67 into the cell cytoplasm has also been reported for cancer cells cultured as MCCs [57]. Such study proved that cytosolic Ki67 was not a proliferative marker and suggests a time-dependent proliferative activity of our ALI multilayered co-cultures. The biochemical mechanisms triggering and regulating membranous/cytoplasmic Ki67 translocation and its cellular effects, remain unknown. Our data leads to the hypothesis that the changes in Ki67 protein localization may be somehow linked to the increasing cell death of ALI multilayered co-cultures at 7 and 14 days, as shown in Fig. 2c. This result can find some correspondence into previous studies, showing that co-culturing of A549 cells with MRC-5 cells does not induce a positive increase in cell survival [17, 51, 58]. Contradictory results were obtained between cell viability and ATP levels (Fig. 2c). This was associated to the fact that, while cell viability was measured on A549 cells isolated from the cultures, ATP levels were measured for ALI MCCs as a whole, keeping into account also the MRC-5 component.

Similarly to ALI multilayered mono-cultures [19], the LY permeability data demonstrated that an epithelial barrier was formed at 24 h in ALI multilayered co-cultures and that it remained intact over 14 d in culture (Fig. 2c). Interestingly, the permeability of ALI multilayered co-cultures remained unaltered even when the percentage of live A549 cells decreased (at 7 and 14 d). We believe this could be associated with the increased thickness of the MCCs at these time-points, as well as to the additional layers formed by MRC-5 cells on the basolateral side of the cultures. The formation of multilayers of MRC-5 cells was sought in our study, as this cell line is constituted by human embryonic (diploid) lung fibroblasts, which have been reported to be able to escape contact-mediated growth inhibition [59].

As in the case of ALI multilayered mono-cultures [19], the multilayered architecture of the in vitro ALI MCC was not sufficient to guarantee TEER (Fig. 2c). This is consistent with previous reports on in vitro epithelial models formed by A549 cells [60] and is associated to the inability of A549 cells to form functional tight-junctions, as extensively reported in the literature [61, 62].

As previously reported for ALI multilayered mono-cultures [19], in co-cultures E-cadherin (epithelial marker [63]) was expressed at all time-points tested (Fig. 2d). Not surprisingly, the mesenchymal protein vimentin was also expressed in both cell cultures, at all time-points (Fig. 2d). Vimentin is in fact a protein normally expressed in cells of mesenchymal origin, such as the A549 cell line. Fibronectin expression (mesenchymal marker), was absent at 24 h but was detected in both MCCs after 48 h growth (Fig. 2d). Thus, we concluded that, overtime, A549 cells acquired metastatic-like properties independently from the presence of fibroblasts in the culture.

ALI multilayered co-cultures developed MDR, similarly to mono-cultures. Our data clearly demonstrated, in fact, that neither the intrinsic nor the extrinsic apoptotic pathways were activated in ALI multilayered co-cultures as a result of drug exposure (Fig. 3). Following drug exposure, in fact, ALI multilayered co-cultures showed a complete lack in: (i) caspases 1–10 activation, (ii) release of cytochrome c, and (iii) subsequent activation of procaspase-3 and PARP into their active cleaved forms. In addition, Bcl-xl expression was increased in drug-treated cultures, inhibiting the potential activation of any MOMP-triggered signalling cascade.

Notably, although ALI multilayered mono- and co-cultures showed the same MAF score for the MRP1/2 drug efflux pumps (Additional file 2: Figure S6), chemoresistance was higher upon co-culturing of A549 cells with fibroblasts (Fig. 4). In order to validate our data, we proved that no distinct difference could be found among the functional and morphological properties featured by the ALI multilayered mono- and co-cultures tested (Fig. 5). For example, altered metabolism in cancer cells is believed to contribute to chemoresistance. However, drug-resistant cancer cells are generally characterized by higher absolute levels of intracellular ATP [64]. Hence, the increased chemoresistance of ALI multilayered co-cultures could not be explained by the lower ATP levels detected (Fig. 5b). Surfactant-associated proteins (SPs), which are components of the human pulmonary surfactant produced by type II alveolar cells, are one of the barriers to the exertion of the therapeutic effect of drugs in the lung [4]. Thus, increased expression levels of these proteins can induce chemoresistance. Our results proved that SPs expression was not associated to the chemoresistance of ALI multilayered co-cultures (Fig. 5c-d). Although the suitability of A549 cells as in vitro cell model for alveolar type II epithelial cells is questionable [65, 66], literature supports that this cell line can produce SPs [67–69]. Based on the evidence described above, no substantial difference was found in the functional and morphological characteristics of the two in vitro systems that would account for the increased chemoresistance detected in the co-culture model. This proved to be in favor to our hypothesis that the MDR of ALI multilayered co-cultures was due to the establishment of a cancer cell-fibroblast cross-talk.

We suggest that, in ALI multilayered co-cultures, TGF-β1 played a contributing role in the communication between fibroblasts and NSCLC cells, inducing MDR. This conclusion is supported by the fact that the free-active TGF-β1, the ligand that binds TGF-βR and exerts signaling functions, was more abundant in ALI multilayered co-cultures than in mono-cultures (Fig. 6b). Also, this is consistent with the literature showing that biochemical cross-talk in NSCLC is driven to a great extent by cancer-derived growth factors, such as EGF and TGF-β1 [70]. While physical contact was impaired due to the architecture of the in vitro co-culture model, biochemical interactions could in fact occur across the apical to basolateral compartments of our ALI MCCs: in ALI multilayered mono-cultures, for example, TGF-β1 was detected in both apical and basolateral compartments (Fig. 6b). This demonstrated that TGF-β1 was secreted by A549 cells grown on the apical side in an un-polarized manner and was able to diffuse to the basolateral chamber through the PET membrane pores. It also implied that in ALI multilayered co-cultures, the chemical stimuli (i.e., TGF-β1) secreted by cancer (A549) cells could diffuse and reach MRC-5 cells.

Notably, as previously mentioned, the levels of free-active TGF-β1 resulted higher in the supernatants harvested from ALI multilayered co-cultures as compared to those detected in mono-cultures (Fig. 6b). This could be due to: (i) an increased secretion of TGF-β1 from A549 cells upon co-culturing with fibroblasts; (ii) the secretion of TGF-β1 from MRC-5 cells; or (iii) an enhanced enzymatic activity responsible for releasing the free-active TGF-β1 from its inactive precursor molecule (here referred to as “total TGF-β1”). To investigate this further, we measured the levels of total TGF-β1 (Fig. 6b) and found that the levels of total TGF-β1 secreted by ALI multilayered co-cultures were lower in the apical chamber and higher in the basolateral compartment. Although a definitive conclusion could not be drawn, these results seemed to suggest that, upon A549/MRC-5 co-culturing, the enzymatic release of the free-active TGF-β1 was increased in the apical chamber, and MRC-5 cells were able to secrete TGF-β1 in the basolateral compartment.

The establishment of cancer cell-fibroblast cross-talk through TGF-β1 suggested that, in ALI multilayered co-cultures, the normal human immortalized MRC-5 cells may have shifted towards a CAF phenotype. Cancer cells are in fact capable to co-opt normal fibroblasts through cross-talk, thus triggering their trans-differentiation into CAFs [71]. This has been first observed in vivo [70] and it has found evidence also in in vitro studies on co-culture NSCLC models, where the gene expression of normal pulmonary fibroblasts has been shown to be altered overtime [72]. Trans-differentiation is also responsible for MDR in NSCLC [73, 74]. Although various proteins (e.g. α-smooth muscle actin and fibroblast activation protein) have been used to probe CAFs, presently there is not an evident specific marker for this cell type [75]. This prevented us from further investigating the MRC-5 trans-differentiation into CAFs. Our focus was therefore shifted into the effects of TGF-β1-dependent cross-talk on A549-cell biomolecular signature and how it affected their MDR mechanisms.

TGF-β1 is a pleiotropic cytokine that exerts its function on several cell types. The increased TGF-β1 secretion levels detected in ALI multilayered co-cultures could play a role not only in inducing MRC-5-cell trans-differentiation but also in triggering the chemoresistance of A549 cells. In cancer cells, upon the binding of free-active TGF-β1 to the receptor TGF-βR, SMAD2/3 are translocated to the receptor complex and phosphorylated. This induces cancer cells’ survival and drug resistance (Additional file 2: Figure S8). In the A549 cell line, TGF-β1 is known to activate SMAD-2 through phosphorylation, inducing EMT, at doses equal or above 5 ng/ml [76]. However, no phospho-SMAD2 (p-SMAD2) could be detected in A549 cells lysates of untreated (NT) or drug-treated ALI multilayered co-cultures (Fig. 7). Our conclusion was that the co-culture MDR was not mediated through SMAD2/3 signalling cascade.

TGF-β1 can also activate protein kinase B (known as AKT) through TGF-βR-induced phosphatidylinositol-3-kinases (PI3K), which phosphorylates AKT (Additional file 2: Figure S8). Our results suggested that, in ALI multilayered co-cultures, free-active TGF-β1 indeed induced MDR through activation of the PI3K/AKT signalling pathway. When inhibiting the phosphorylation of AKT with perifosine, in fact, docetaxel efficacy was increased, although statistical changes were detected only at one concentration (Fig. 8). This suggested that MDR of ALI multilayered co-cultures was mediated through the PI3K/AKT pathway. Expression and p-MDM2 and up-regulation in the levels of MCL-1 (Fig. 7) further supported our conclusion that, TGF-β1 induced MDR in ALI multilayered co-cultures by activating the PI3K/AKT signalling cascade.

Our data from the western blot analysis strikingly pointed out that p-p53, a marker of apoptosis, was completely depleted from A549 cells forming ALI multilayered co-cultures (Fig. 7). The complete depletion of p-p53 could be associated to the AKT-triggered phosphorylation of MDM2, which was detected in the lysates of A549 cells isolated from ALI multilayered co-cultures (Fig. 7). In apoptosis-defective cells, autophagy prevents death from necrosis, promoting tumour growth and tumorigenesis [77–79]. Based on the apoptosis deficiency found in the A549 cells forming ALI multilayered co-cultures and the expression of p-mTOR (Fig. 7b), we could conclude that MDR was also promoted through an autophagy inhibition. This is in agreement with the scientific literature, reporting that aberrant activation of PI3K/AKT/mTOR pathway is one of the mechanisms of acquired MDR in NSCLC patients [80]. In this instance, it should be noted, the phosphorylation levels of both mTOR and MDM2 were reduced in ALI multilayered mono-cultures, as compared to co-cultures (Fig. 7a). This further proved that the MDR mechanism of ALI multilayered co-cultures was specific to this in vitro model and could have been activated by the cancer cell-fibroblast cross-talk through an increased secretion/activation of TGF-β1.

Phosphorylation of mTOR could also be triggered by the K-RAS mutation expressed by the A549 cell line [81], as both PI3K/AKT and MEK/ERK signalling regulate the mTOR axis (Additional file 2: Figure S8). In this instance, we could exclude that the phosphorylation of mTOR detected in drug-treated ALI multilayered co-cultures was solely triggered by the K-RAS mutation. As described above, inhibiting AKT activation by perifosine was capable of restoring docetaxel efficacy (Fig. 8), suggesting that AKT was actively involved in inducing mTOR phosphorylation. To further corroborate our conclusion, expression of survivin, a protein of the inhibitor of apoptosis (IAP) family, was not found in lysates of A549 cells forming ALI multilayered co-cultures (Fig. 7). Survivin inhibits apoptosis following exposure to cytotoxic drugs [82]. Its expression correlates with expression of cyclooxygenase-2 (COX2) in NSCLC [83] (Additional file 2: Figure S8). Oncogenic K-RAS regulates proliferation and cell functions in lung epithelial cells through induction of COX2 [84, 85]. Therefore, the lack of survivin expression in ALI multilayered co-cultures proved that the K-RAS mutation of A549 cells was not the main mechanism driving the MDR of our in vitro co-culture model.

Interestingly, our data showed an increase in MDR features following exposure to anti-cancer drugs. In detail, activation of the PI3K/AKT/mTOR pathway was not the only signalling cascade inducing MDR in ALI multilayered co-cultures. When inhibiting the phosphorylation of mTOR by rapamycin exposure, the expression of cIAP-1/2 increased in drug-treated ALI multilayered co-cultures, as compared to the untreated models (Fig. 9). cIAP-2 is known to be upregulated in NSCLC [86], whereas chemotherapy induces cIAP-1 up-regulation [87], suggesting an important adaptive role of these two proteins in response to anti-cancer drugs treatments. Consistent with the literature, with the expression of cIAP-1/2 we observed no increase in docetaxel efficacy, over a wide range of drug concentrations in the presence of rapamycin (0.1, 1 and 100 μM). Based on our data, we suggest that the lack in higher docetaxel efficacy in the presence of rapamycin was due, on one side, to the induction of the MEK/ERK signalling pathway and, on the other, by up-regulation in cIAP-1/2 expression, through p-AKT signalling. Other groups have in fact reported that, in cancer cells as well as in cancer specimens, mTOR inhibition with rapamycin induces feedback activation of the AKT survival pathways [88, 89]. This mechanism does indeed support the cIAP-1/2 up-regulation detected in our ALI multilayered co-cultures exposed to rapamycin.

## Conclusions

In the present study, we developed an advanced in vitro 3D model capable to (i) reproduce some of the TME-triggered pathways promoting NSCLC progression and chemoresistance, and (ii) enable the testing of inhaled anti-cancer drugs administered via aerosol. Our results describe for the first time an ALI multilayered co-culture formed by growing NSCLC cells with lung fibroblasts on Transwell™ supports. The in vitro model developed was characterized by a mesenchymal phenotype and MultiDrug Resistance (MDR), which was significantly higher than that found in an ALI multilayered mono-culture of NSCLC cells. The fibroblasts showed to influence the MDR mechanism of the in vitro model, by triggering activation of the PI3K/AKT/p-mTOR signalling cascade via TGF-β1 secretion. Interestingly, this was not the only signalling cascade inducing MDR in ALI multilayered co-cultures, as mTOR inhibition with rapamycin induced feedback activation of the AKT survival pathways.

The knowledge developed in our study provides a step forward towards the establishment of an in vitro tool capable of guiding the rational selection of inhaled anti-cancer candidates, thus minimizing the number of animals used *per* study (principle of Reduction, “3Rs” framework) in the future. Current preclinical studies on inhaled compounds rely mainly on small animal models (particularly rodents) [90], which do not mimic the anatomy of the human respiratory tract [91]. Besides differences in the gross architecture of the airways, animal models and humans differ also in the amount of mucus in the airways and in the distribution and types of surface epithelial cell populations lining the airways. It has been shown that the average number of cells per alveolus for rats versus humans is: 21 vs. 1481 for endothelial cells, 13 vs. 106 for interstitial cells, 6 vs. 67 for epithelial type II cells, 4 vs. 40 for epithelial type I cells, and 1.4 vs. 12 for alveolar macrophages [92]. Even the most advanced class of in vivo preclinical models, namely the patient-derived xenograft model, displays limitations due to the rapid replacement of the original human stroma environment by murine components [93]. Therefore, there is often a lack of correlation between animal experiments and human trials. This is particularly evident in how different species respond to drug therapy, and is a major contributor to the high rate of failure (approximately 90–95%) in anti-cancer drug discovery [94].

The tissue-mimetic model described in the present study has demonstrated to: (i) incorporate biological complexity (3D architecture and multicellularity), (ii) achieve a “closer relevancy to the patient model” by reproducing MDR mechanisms and feedback activation signalling processes observed in human NSCLC patients, and (iii) integrate culturing conditions at the Air-Liquid Interface that are compatible with aerosol administration methods. The global valence of the presented preclinical model is its applicability as a valid alternative to animal-based inhalation studies in NSCLC research and in the regulatory field. Its use ranges from the efficacy screening of aerosolized chemotherapy and nano-enabled drug delivery systems, to testing the hazard posed by airborne pollution. Future studies will investigate the integration of our in vitro platform with several other key factors triggering chemoresistance in NSCLC patients, such as genetic variability, extracellular matrix components and immune-competence.

## Additional files

Additional file 1: **Video S1.** Animated reconstruction of Z-stack LSCM images of ALI multilayered co-cultures grown for 72 h and stained with: Hoechst 33342 for cell nuclei (in blue), rhodamine phalloidin for F-actin (in red) and Ki67 protein expression (in green). Objective lens, 63 × . (AVI 14178 kb)

Additional file 2: **Figure S1.** Representative LSCM images of the (from left to right) F-actin organi-zation (in red) and Ki67 pro-tein expression (in green) in ALI multilayered co-cultures grown for 24 h, 48 h, 72 h, 7 d and 14 d. **Figure S2.** Representative LSCM images of ALI multilayered co-cultures grown for 24 h, 48 h, 72 h, 7 d and 14 d and stained with Hoechst 33342 for cell nuclei (in blue) and EthD-1 for dead cells (in red). **Figure S3.** Scatter plots showing the percentage (%) of live and dead A549 cells detected in (A) ALI multilayered mono- and (B) co-cultures by means of flow cytometry. **Figure S4.** Chemoresistance of ALI multilayered co-cultures – Direct inoculation vs nebuli-zation. **Figure S5.** Western blot analysis of lysates of A549 cells grown in (A) ALI multilayered mono-cultures and (B) ALI multilayered co-cultures that were exposed to docetaxel (Doc), vinblastine (Vin), cytarabine (Cyt) or methotrexate (Met) at their nominal IC50 for 72 h. **Figure S6.** Representative histograms resulting from the MDR assay carried out on ALI mul-tilayered mono- (top) and co- (bottom) cultures. **Figure S7.** Western blot analysis of phospho-SMAD2 (p-SMAD2) expression in A549 cells cultured as sub-confluent mono-cultures on plastic substrates. **Figure S8.** Schematics of how grow factors (HGF, TGF-β1 and EGF) can induce MDR in NSCLC cells. **Figure S9.** Expression levels of p-mTOR in A549 cells grown in ALI multilayered mono-cultures (black bars) and ALI multilayered co-cultures (grey bars) that were exposed to docetaxel (Doc), vinblastine (Vin), cytarabine (Cyt) or metho-trexate (Met) at their nominal IC50 for 72 h. **Table S1.** Co-localization of Ki67 protein expression and nuclear staining in ALI multi-layered co-cultures. (PDF 1274 kb)

## Data Availability

All data generated or analysed during this study are included in this article. The raw datasets generated during and/or analysed during the current study are available from the corresponding author on reasonable request.

## Abbreviations

ALI: Air-Liquid Interface
ATP: Adenosine TriPhosphate
CAF: Cancer-associated fibroblast
ELISA: Enzyme-Linked ImmunoSorbent Assay
IAP: Inhibitor of apoptosis protein
LY: Lucifer Yellow
mAb: Monoclonal antibody
MCC: Multilayered Cell Culture
MDR: MultiDrug Resistance
NSCLC: Non-Small-Cell Lung Cancer
PBS: Phosphate Buffered Saline
TEER: TransEpithelial Electric Resistance
TME: Tumour MicroEnvironment
