# Supplementary material for: ALI multilayered co-cultures mimic biochemical mechanisms of the cancer cell-fibroblast cross-talk involved in NSCLC MultiDrug Resistance
Source: BMC Cancer. 2019 Aug 29;19:854. doi: 10.1186/s12885-019-6038-x (PMC6714313; doi:10.1186/s12885-019-6038-x)
Supplement: Supplementary file 2 — Figure S1. Representative LSCM images of the (from left to right) F-actin organi-zation (in red) and Ki67 pro-tein expression (in green) in ALI multilayered co-cultures grown for 24 h, 48 h, 72 h, 7 d and 14 d. Figure S2. Representative LSCM images of ALI multilayered co-cultures grown for 24 h, 48 h, 72 h, 7 d and 14 d and stained with Hoechst 33342 for cell nuclei (in blue) and EthD-1 for dead cells (in red). Figure S3. Scatter plots showing the percentage (%) of live and dead A549 cells detected in (A) ALI multilayered mono- and (B) co-cultures by means of flow cytometry. Figure S4. Chemoresistance of ALI multilayered co-cultures – Direct inoculation vs nebuli-zation. Figure S5. Western blot analysis of lysates of A549 cells grown in (A) ALI multilayered mono-cultures and (B) ALI multilayered co-cultures that were exposed to docetaxel (Doc), vinblastine (Vin), cytarabine (Cyt) or methotrexate (Met) at their nominal IC50 for 72 h. Figure S6. Representative histograms resulting from the MDR assay carried out on ALI mul-tilayered mono- (top) and co- (bottom) cultures. Figure S7. Western blot analysis of phospho-SMAD2 (p-SMAD2) expression in A549 cells cultured as sub-confluent mono-cultures on plastic substrates. Figure S8. Schematics of how grow factors (HGF, TGF-β1 and EGF) can induce MDR in NSCLC cells. Figure S9. Expression levels of p-mTOR in A549 cells grown in ALI multilayered mono-cultures (black bars) and ALI multilayered co-cultures (grey bars) that were exposed to docetaxel (Doc), vinblastine (Vin), cytarabine (Cyt) or metho-trexate (Met) at their nominal IC50 for 72 h. Table S1. Co-localization of Ki67 protein expression and nuclear staining in ALI multi-layered co-cultures. (PDF 1274 kb) [file 12885_2019_6038_MOESM2_ESM.pdf]

ALI multilayered co-cultures mimic biochemical mechanisms of the cancer cell-fibroblast cross-talk involved in NSCLC MultiDrug Resistance

Dania Movia[1,*], Despina Bazou[2], Adriele Prina-Mello[1,3]

[1]Department of Clinical Medicine/Trinity Translational Medicine Institute (TTMI), Trinity College Dublin, Ireland

[2]Mater Misericordiae Hospital, Dublin, Ireland

[3]AMBER Centre, CRANN Institute, Trinity College Dublin, Ireland

- Supporting Information -

SUPPORTING FIGURES

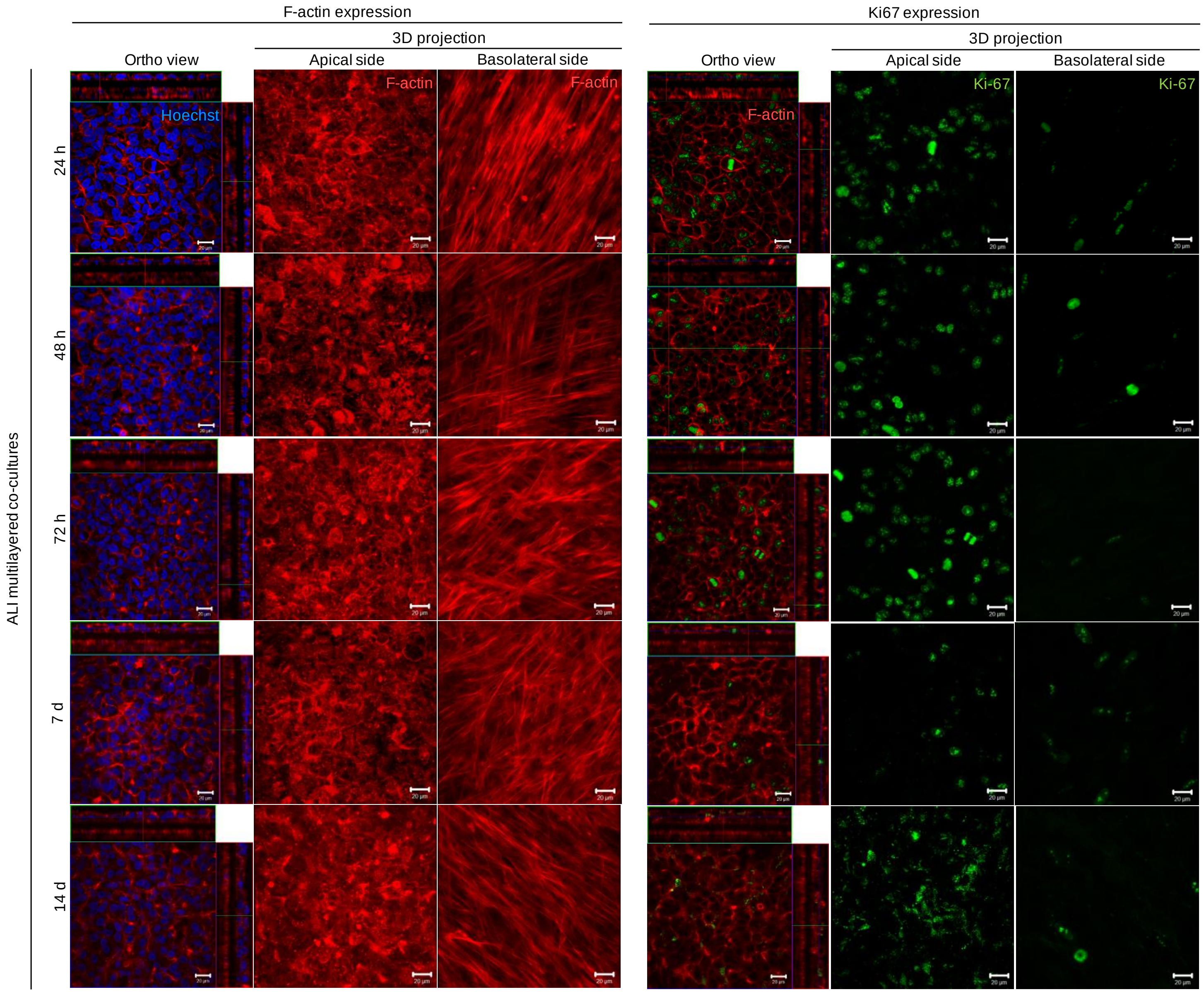

Figure S1 Representative LSCM images of the (from left to right) F-actin organization (in red) and Ki67 protein expression (in green) in ALI multilayered co-cultures grown for 24 h, 48 h, 72 h, 7 d and 14 d. Cell nuclei were also stained with Hoechst 33342 (in blue). Z-stack images were acquired and are here presented in orthogonal view (ortho view) or as three-dimensional (3D) projections. Images of the apical (formed by A549 cells) and basolateral side (MRC-5 cells) of the Transwell™ supports are reported. Scale bars: 20 μm (objective lens, 63×).

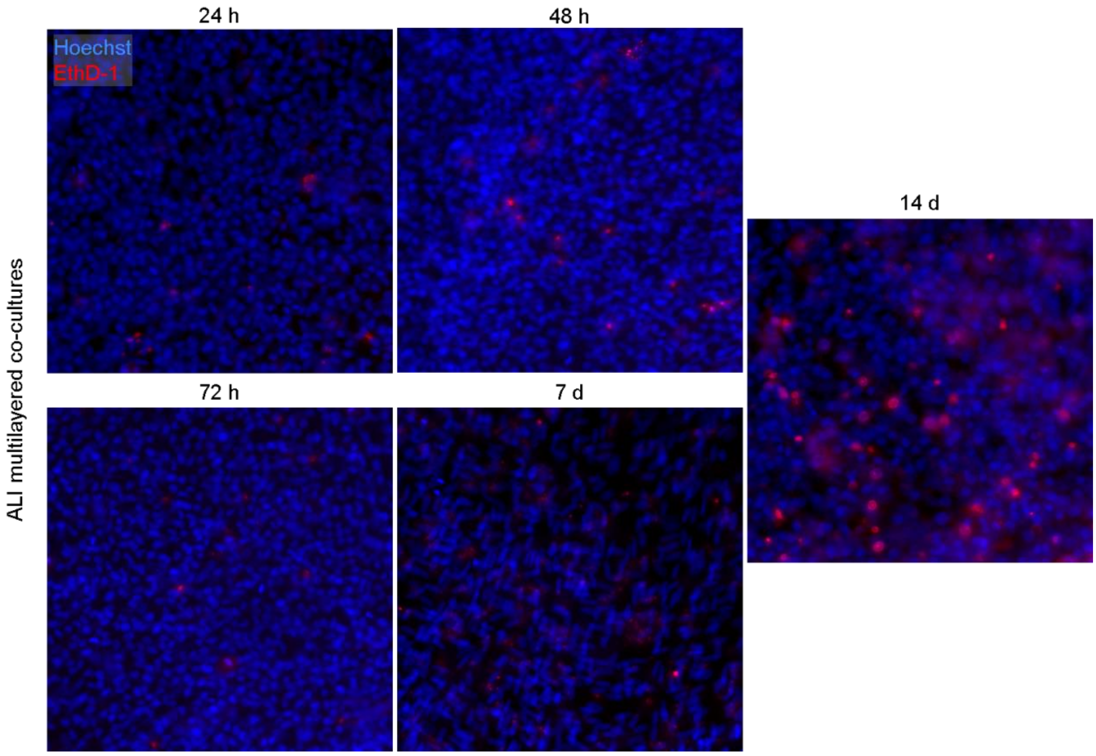

Figure S2. Representative LSCM images of ALI multilayered co-cultures grown for 24 h, 48 h, 72 h, 7 d and 14 d and stained with Hoechst 33342 for cell nuclei (in blue) and EthD-1 for dead cells (in red). Z-stack images were acquired and are here presented in projection mode. Objective lens, 40×.

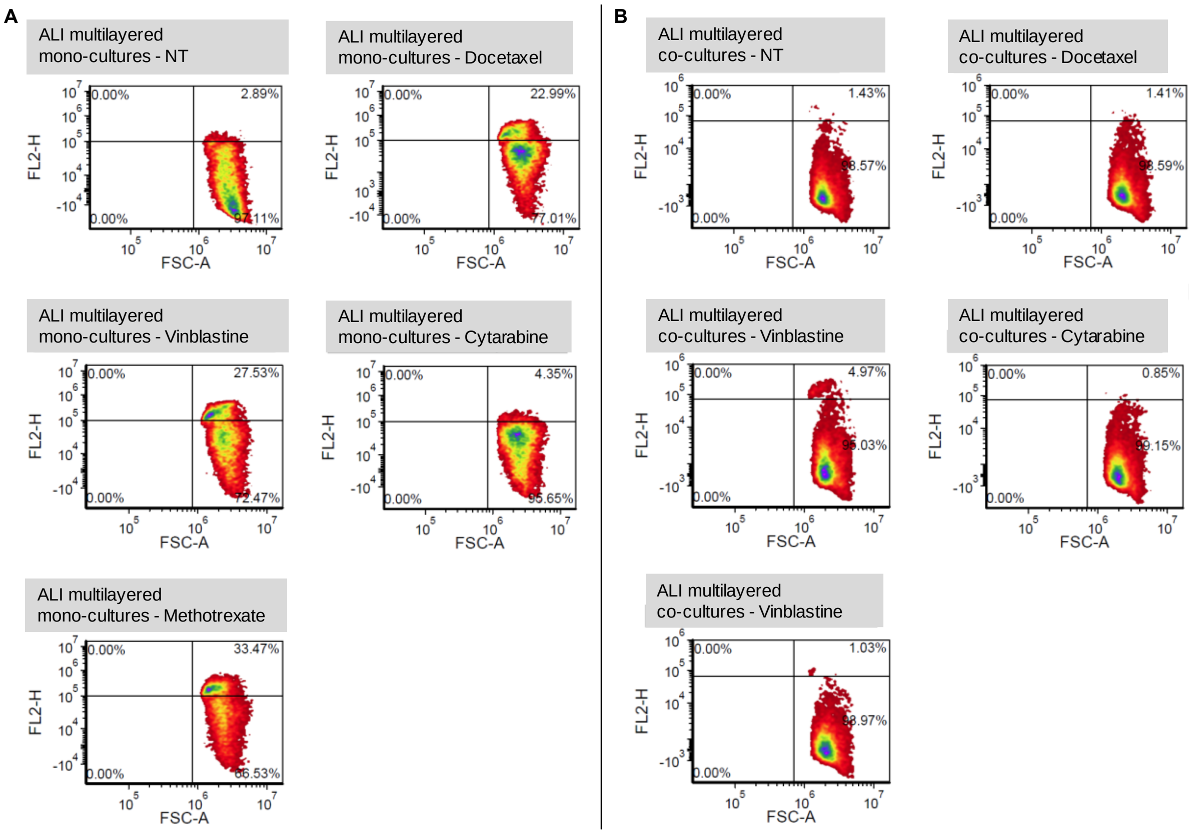

Figure S3. Scatter plots showing the percentage (%) of live and dead A549 cells detected in (A) ALI multilayered mono- and (B) co-cultures by means of flow cytometry. MCCs were exposed, by direct inoculation, to four different chemotherapeutics (docetaxel, vinblastine, cytarabine and methotrexate) at their nominal IC_{50} concentration for 72 h. Untreated MCCs were also analysed as negative control (NT). Counted live and dead cells can be visualised in the bottom right and top right quadrants, respectively. Data shown are representative of the results collected for multiple replicates run in the study.

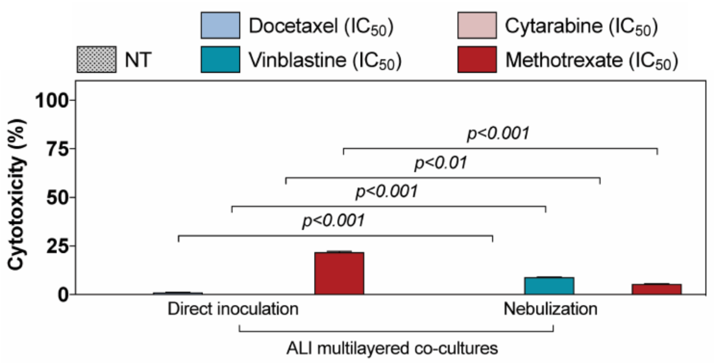

Figure S4. Chemoresistance of ALI multilayered co-cultures – Direct inoculation *vs* nebulization. Cytotoxicity detected in ALI multilayered co-cultures exposed to four anti-cancer drugs (docetaxel, cytarabine, vinblastine and methotrexate) at their nominal IC_{50} concentration for 72 h, by direct inoculation or nebulization. Data are reported as average ± standard error of the mean ($n_{replicates}$ = 2; n_{tests} = 3). *p* values indicate significant differences (two-way ANOVA and Bonferroni post-test).

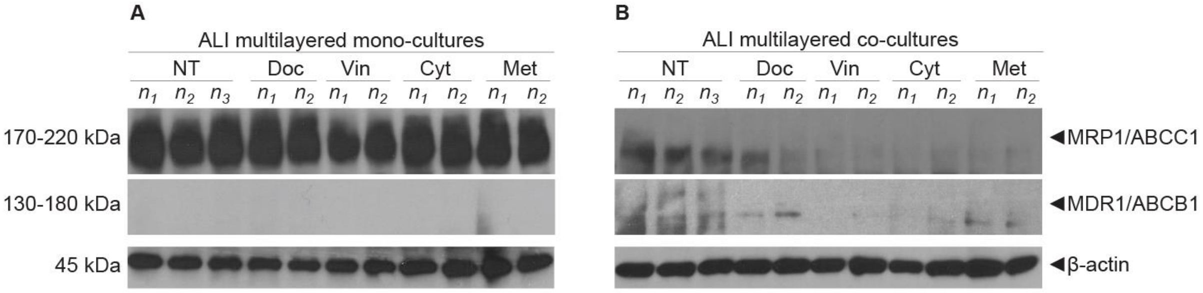

Figure S5. Western blot analysis of lysates of A549 cells grown in (A) ALI multilayered mono-cultures and (B) ALI multilayered co-cultures that were exposed to docetaxel (Doc), vinblastine (Vin), cytarabine (Cyt) or methotrexate (Met) at their nominal IC_{50} for 72 h. Drugs were administered by direct inoculation. Untreated cultures (NT) were also analysed for comparison. The expression levels of MRP1/ABCC1 and MDR1/ABCB1 proteins are shown. Abbreviations “n_1”, “n_2” and “n_3” indicate different biological replicates. β-actin expression levels are also reported as protein loading controls.

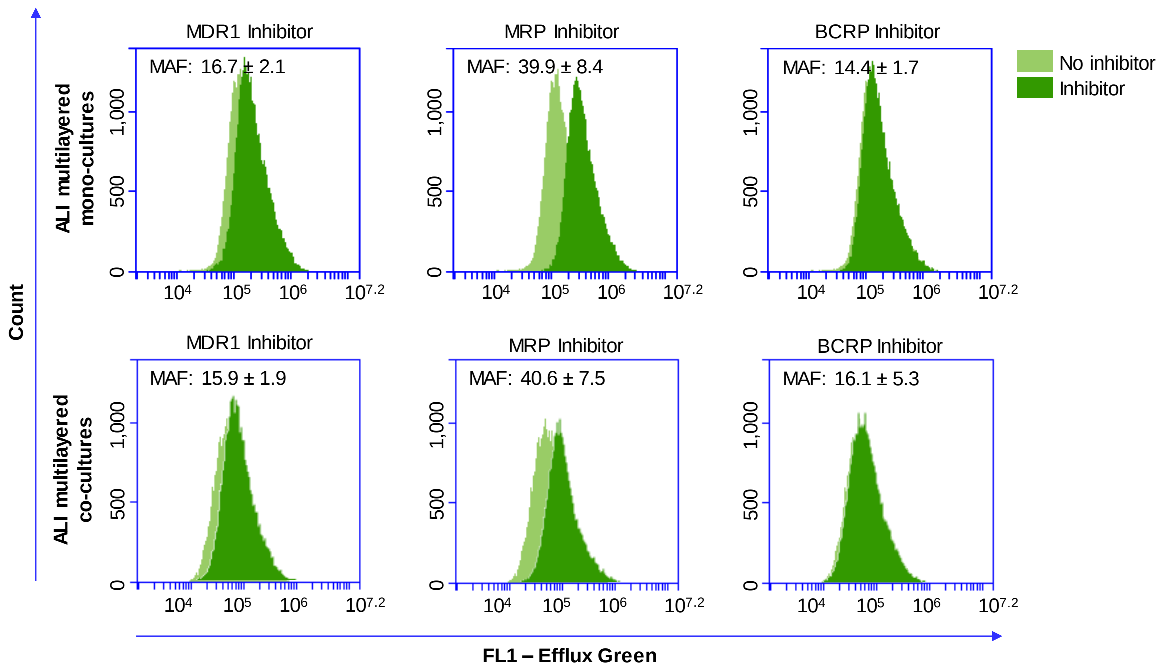

Figure S6. Representative histograms resulting from the MDR assay carried out on ALI multilayered mono- (top) and co- (bottom) cultures. Live A549 cells isolated from the ALI MCCs were incubated with Efflux Green Detection Reagent in the presence or absence of specific inhibitors for MDR1, MRP1/2 or BCRP transporters, according to the assay protocol. Resulting fluorescence was measured using flow cytometry. Dark-green histograms show fluorescence of inhibitor-treated samples and light-green histograms show fluorescence of untreated cells. Differences in fluorescence intensity in the presence of an inhibitor are indicative of a corresponding transporter activity. The numbers in the upper left corners are the MAF (multidrug resistance activity factors) scores extrapolated from the data analysis. MAF values are reported as average ± standard error of the mean ($n_{replicates} = 3$; $n_{tests} \geq 3$).

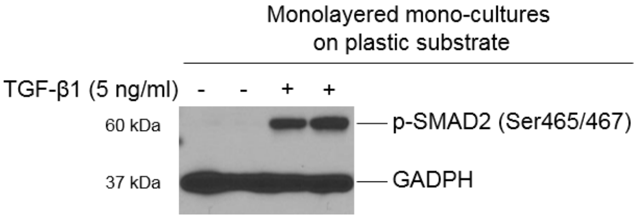

Figure S7. Western blot analysis of phospho-SMAD2 (p-SMAD2) expression in A549 cells cultured as sub-confluent mono-cultures on plastic substrates. After 24 h in culture, A549 cells were stimulated with human recombinant TGF-β1 (5 ng/ml) (Cell Signaling Technology Inc, Brennan & Company, Ireland) for 24 h. TGF-β1 was reconstituted in sterile 20 mM citrate (Cell Signaling Technology Inc, Brennan & Company, Ireland) and then diluted at de-

sired concentration in supplemented DMEM medium. Concentration tested was based on existing scientific literature describing the response of A549 cell line to TGF-β1 [1]. Expression levels in untreated cultures were also analysed for comparison. The expression of GADPH is reported as a protein loading control.

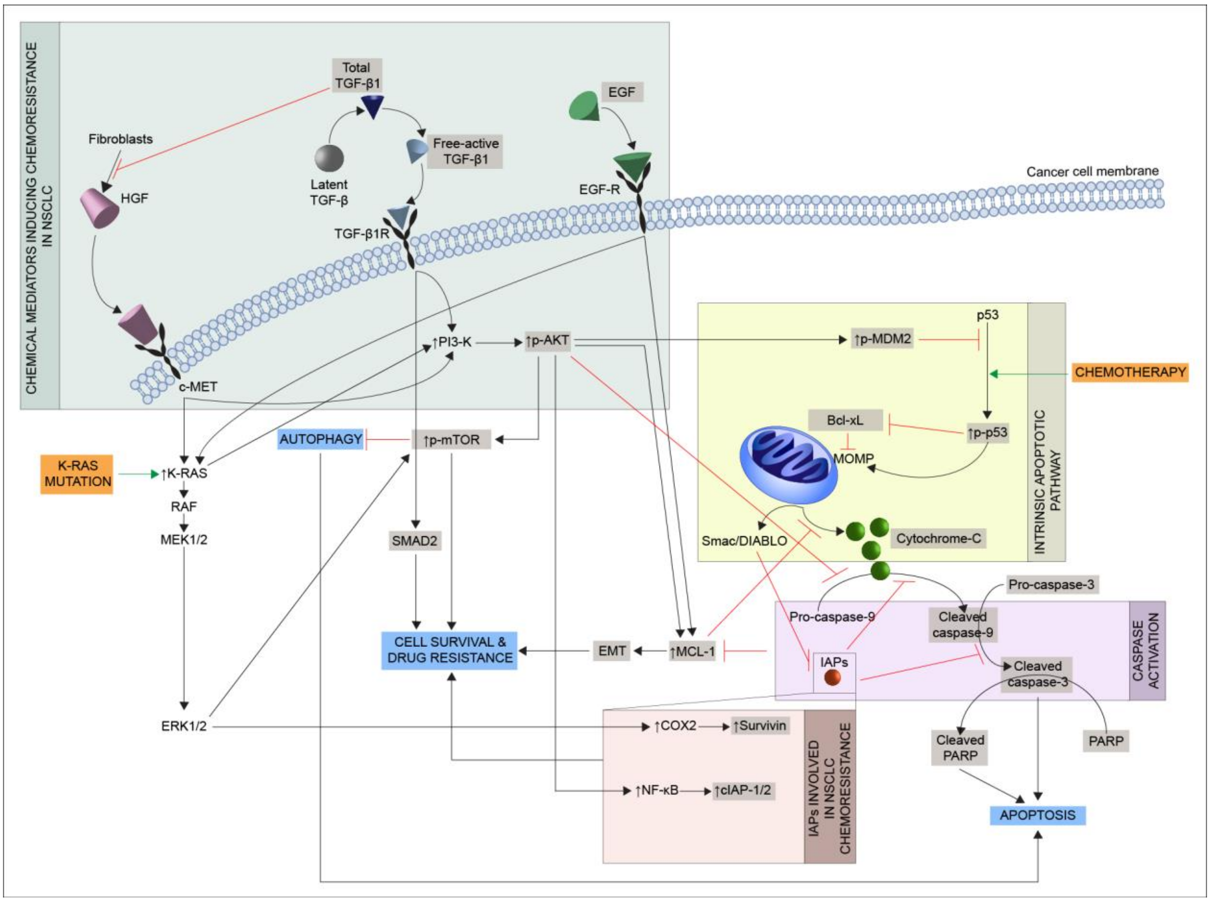

Figure S8. Schematics of how grow factors (HGF, TGF-β1 and EGF) can induce MDR in NSCLC cells. Grey boxes highlight the biomolecular molecules analysed in this study. Please note that, the secretion of hepatocyte growth factor (HGF) by MRC-5 cells as chemical stimuli capable of inducing fibroblasts trans-differentiation was not examined within this study, as TGF-β1 is reported to be a potent inhibitor of its secretion [2]. Abbreviations: COX2, cyclooxygenase-2; EGF, epidermal growth factor; EGF-R, epidermal growth factor receptor; EMT, epithelial-to-mesenchymal transition; HGF, hepatocyte growth factor; TGF-β, transforming growth factor-beta; TGF-β1R, transforming growth factor-beta1 receptor.

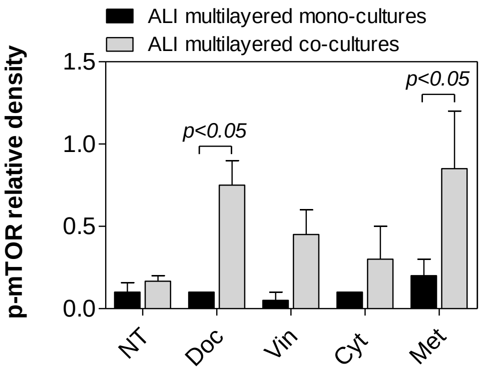

Figure S9. Expression levels of p-mTOR in A549 cells grown in ALI multilayered mono-cultures (black bars) and ALI multilayered co-cultures (grey bars) that were exposed to docetaxel (Doc), vinblastine (Vin), cytarabine (Cyt) or metho-trexate (Met) at their nominal IC50 for 72 h. Drugs were administered by direct inoculation. Untreated cultures (NT) were also analysed for comparison. p-mTOR expression levels were detected by Western blotting and are reported as relative density, *i.e.* the ratio between p-mTOR and β-actin used as protein loading control. Data are reported as average ± standard error of the mean ($n_{replicates} \geq 2$). $p<0.05$ indicates a significant difference (two-way ANOVA with Bonferroni post-test).

SUPPORTING TABLES

Table S1. Co-localization of Ki67 protein expression and nuclear staining in ALI multi-layered co-cultures. MCCs were grown for various culturing time-points and then stained and imaged, as described in the material and methods section. The co-localization study was carried out on representative Z-planes of LSCM images by means of the ZEN software (ZEISS, Germany), setting an intensity threshold equal to 50 for both channels. An overlap coefficient equal to 1 indicates perfect co-localization of the Ki67 protein with cell nuclei, while no co-localization corresponds to an overlap coefficient of 0.

**Time-point**	**Overlap coefficient**
	**ALI multilayered co-cultures**
24 h	0.70
48 h	0.68
72 h	0.74
7 d	0.68
14 d	0.48

SUPPORTING REFERENCES

1. Kasai H, Allen JT, Mason RM, Kamimura T, Zhang Z: **TGF-beta1 induces human alveolar epithelial to mesenchymal cell transition (EMT)**. *Respir Res* 2005, **6**(1):56.
2. Gohda E, Matsunaga T, Kataoka H, Yamamoto I: **TGF-β is a potent inhibitor of hepatocyte growth factor secretion by human fibroblasts**. *Cell biology international reports* 1992, **16**(9):917-926.
